# *C*-Glucosylation as a tool for the prevention of PAINS-induced membrane dipole potential alterations

**DOI:** 10.1038/s41598-021-83032-3

**Published:** 2021-02-24

**Authors:** Ana Marta de Matos, Maria Teresa Blázquez-Sánchez, Carla Sousa, Maria Conceição Oliveira, Rodrigo F. M. de Almeida, Amélia P. Rauter

**Affiliations:** 1 Centro de Química Estrutural, Faculdade de Ciências, Universidade de Lisboa, Ed. C8, Campo Grande, 1749-016 Lisboa, Portugal; 2 Facultad de Ciencias y Artes, Universidad Católica Santa Teresa de Jesús de Ávila (UCAV), 05005 Avila, Spain; 3 Centro de Química Estrutural, Instituto Superior Técnico, Mass Spectrometry Facility, Av. Rovisco Pais, 1049-001 Lisboa, Portugal

**Keywords:** Biophysics, Chemistry

## Abstract

The concept of Pan-Assay Interference Compounds (PAINS) is regarded as a threat to the recognition of the broad bioactivity of natural products. Based on the established relationship between altered membrane dipole potential and transmembrane protein conformation and function, we investigate here polyphenols' ability to induce changes in cell membrane dipole potential. Ultimately, we are interested in finding a tool to prevent polyphenol PAINS-type behavior and produce compounds less prone to untargeted and promiscuous interactions with the cell membrane. Di-8-ANEPPS fluorescence ratiometric measurements suggest that planar lipophilic polyphenols—phloretin, genistein and resveratrol—act by decreasing membrane dipole potential, especially in cholesterol-rich domains such as lipid rafts, which play a role in important cellular processes. These results provide a mechanism for their labelling as PAINS through their ability to disrupt cell membrane homeostasis. Aiming to explore the role of *C*-glucosylation in PAINS membrane-interfering behavior, we disclose herein the first synthesis of 4-glucosylresveratrol, starting from 5-hydroxymethylbenzene-1,3-diol, via *C*-glucosylation, oxidation and Horner-Wadsworth-Emmons olefination, and resynthesize phloretin and genistein *C*-glucosides. We show that C-glucosylation generates compounds which are no longer able to modify membrane dipole potential. Therefore, it can be devised as a strategy to generate bioactive natural product derivatives that no longer act as membrane dipole potential modifiers. Our results offer a new technology towards rescuing bioactive polyphenols from their PAINS danger label through C–C ligation of sugars.

## Introduction

PAINS were originally described by Baell & Holloway^1^ as promiscuous molecules capable of interfering with high-throughput screening results, either by behaving as metal chelators, by perturbing membranes, or by non-specific interactions with proteins, amongst other relevant phenomena. Many of these compounds are natural products, among which genistein, resveratrol, epigallocatechin gallate (EGCG), quercetin or curcumin stand out for having been widely studied as potential therapeutic agents against a number of pathophysiological processes and conditions, including cancer^2–4^, inflammation^5^, diabetes^6^, and Alzheimer’s disease^7–10^. Confusing and contradictory results in clinical trials have been putting a question mark on the real therapeutic usefulness of these natural molecules^11–16^. However, while EGCG, quercetin and related compounds displaying catechol or hydroquinone groups are able to covalently inhibit protein activity upon autoxidation in enzyme inhibition assays (which might promptly justify their promiscuity)^1^, resveratrol and genistein lack such reactive moieties in their structure and thus ought to engage in much more subtle alterations in protein structure and conformation. In fact, being very planar and lipophilic molecules, they are believed to act as membrane-perturbing agents^17^. Nevertheless, both compounds have been tested in cell-free assays and found to be effective inhibitors of key enzymes such as BACE-1^18^, DPP-4^19^, PTP1B^20^, α-glucosidase^21^, 6-phosphofructo-1-kinase^22^, COX-1^23^, PDEs^24^, among others. In this perspective, we reckon that new tools can be developed towards the conversion of these and other valuable scaffolds into safe and effective therapeutic lead molecules, with a reliable transposition of bioactivity from primary screening and pre-clinical assays to the final clinical stages, if the reason behind their odd behavior can be clarified and related to their PAINS-nature.

Several authors have reported capacity of these types of compounds to modulate cell signaling pathways leading to desirable therapeutic outcomes in vivo^25–28^. Henceforth, the described affinity of these compounds towards the so-called lipid rafts–which consist of more ordered domains, cholesterol and sphingomyelin (SM)-enriched, within the fluid bilayer enriched in unsaturated phosphatidylcholine (PC) ^29,30^–definitely calls for further investigation in model membrane systems contemplating membrane lateral heterogeneity. Indeed, lipid rafts are known to be related to membrane functionality by playing a crucial role in the regulation of membrane protein activity, protein and lipid trafficking, and, ultimately, signal transduction^31^. On the other hand, the membrane dipole potential is known to exert a crucial part in membrane permeability and ion transport, lipid-protein interactions, regulation of protein conformation and function, among other important roles^32–35^. Together with the transmembrane potential, the boundary potential is a key component of the electric profile associated with cell membranes, and can be subdivided into two subcomponents: the surface potential and the membrane dipole potential^32^. While surface potential is particularly related to the charges at the surface of the membrane^36^, membrane dipole potential results from the relative orientation between the electric dipoles of lipid headgroups and membrane-adsorbed water molecules^37^.

On the basis of some of our previous work^35,38^, we were encouraged to investigate if *C*-glucosylation of polyphenols exhibiting membrane-related PAINS behavior generates new structures devoid of this PAINS feature. Even though *O*-glucosylated compounds are much easier to synthesize, they are highly susceptible to hydrolysis in the gut, and therefore reach their targets as aglycones when administered orally–which should not occur with the chemically/enzymatically stable *C*-glucosyl derivatives. In this work, we tested this hypothesis using 1-palmitoyl-2-(3-{4-[(1*E*,3*E*,5*E*)-6-phenylhexa-1,3,5-trien-1-yl]phenyl}propanoyl)-*sn*-glycero-3-phosphocholine (DPH-PC) fluorescence anisotropy and (*E*)-3-(4-{2-[6-(dioctylamino)naphthalen-2-yl]vinyl}pyridin-1-ium-1-yl)propane-1-sulfonate (di-8-ANEPPS) fluorescence ratiometric measurements to evaluate different membrane physical parameters, namely the dipole potential, in large unilamellar vesicles (LUV) with diverse lipid compositions and phase behavior: **(a)** pure 1-palmitoyl-2-oleoyl-PC (POPC)–representative of the liquid disordered (*l*_d_) lipid phase; **(b)** POPC and cholesterol 1:1–as a model system for the liquid ordered (*l*_o_) phase; and **(c)** a ternary mixture of POPC, cholesterol and *N*-palmitoylsphingomyelin (PSM) 1:1:1–where the *l*_d_ phase coexists with *l*_o_ domains corresponding to lipid rafts^39^. All the lipid proportions indicated are molar ratios.

**Figure 1:**
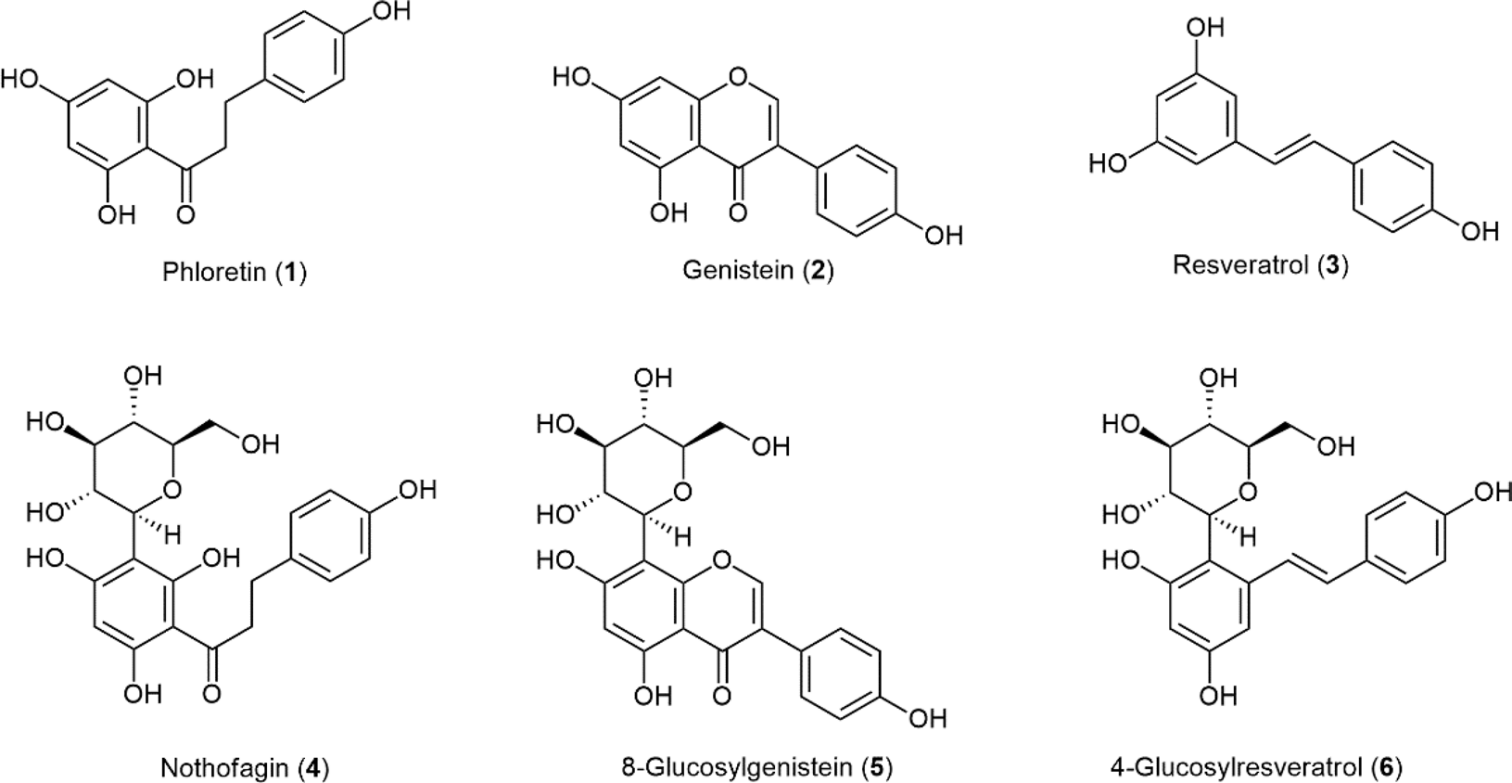
Polyphenols and *C*-glucosyl derivatives in study [structure representation of flavonoids according to The Nomenclature of Flavonoids (IUPAC Recommendations 2017)]^42^. The systematic name of 4-glucosylresveratrol (**6**) is (*E*)-4-(β-d-glucopyranosyl)-5-(4-hydroxystyryl)benzene-1,3-diol and 8-(β-d-glucopyranosyl)genistein is abbreviated as 8-glucosylgenistein.

Three polyphenols were investigated (Fig. 1): phloretin (**1**)–which has been extensively used in membrane-interaction studies including dipole potential measurements^35^, genistein (**2**) and resveratrol (**3**)–two well-known PAINS with broad bioactivity and membrane-interfering behavior^1^.

Whereas the synthesis of the first two *C*-glucosyl derivatives, nothofagin (**4**)^38^ and 8-glucosylgenistein (8G, **5**)^41^, have been previously reported by our group and replicated for this study, we present herein the first synthesis of 4-glucosylresveratrol (E)-4-(β-d-glucopyranosyl)-5-(4-hydroxystyryl)benzene-1,3-diol, **6**]. With this work, we ultimately aim to explore and rationalize the potential mechanisms behind the membrane-interfering behavior of PAINS, while elucidating the role of the sugar moiety in the impairment of such events.

## Results and discussion

### Synthesis of 4-glucosylresveratrol

**Scheme 1:**
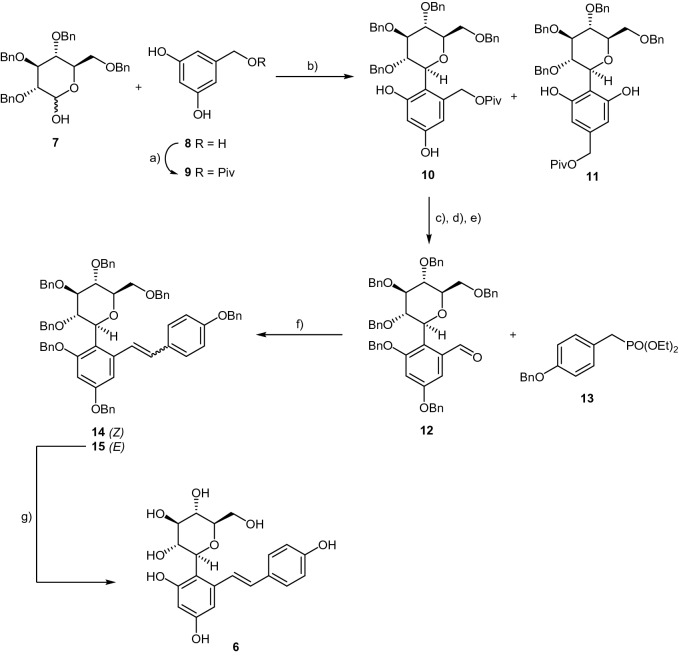
Synthesis of 4-glucosylresveratrol, **6**. Reagents and conditions: (**a**) PivCl, pyridine, 0 °C to r.t., 20 h, 62% yield; (**b**) TMSOTf, drierite, DCM/CH_3_CN, −40 °C to r.t., 43% global yield for both isomers and 24% isolated yield for compound **10**; (**c**) K_2_CO_3_, BnBr, DMF, 0 °C to r.t., 1.5 h, 85% yield; (**d**) LiOH, MeOH/H_2_O, reflux, 36 h, 68% yield; (**e**) PCC, DCM, r.t., 3 h, 81% yield; (**f**) *tert-*BuOK, DMF, 0 °C to r.t., 2 h, 60% global yield for both isomers and 42% isolated yield for the (*E*)-isomer **15**; (**g**) DCM, BCl_3_,-78 °C, 2 h, 35% yield.

Due to the highly conjugated nature of resveratrol, direct *C*-glucosylation of this compound is very challenging and, in our experiments, proved to be unfruitful. In fact, several strategies were thoroughly explored before this goal could eventually be accomplished. One of the most robust approaches attempted was based on direct coupling of 2,3,4,6-tetra-*O*-benzyl-d-glucono-1,5-lactone to 2-bromoresveratrol. However, the anomeric C–C coupling of the gluconolactone and the brominated resveratrol in the presence of butyllithium at − 78 °C gave a complex mixture and did not lead to the desired product. We have then explored a new synthetic pathway, which culminated in the first synthesis of (4-glucosylresveratrol, **6**), presented in Scheme 1. C–C coupling was accomplished via a TMSOTf-promoted Fries-type reaction of the commercially available glucosyl donor **7** and the pivaloyl-protected phenol **9**. The regioisomers **10** and **11** were obtained in 43% global yield, and further purification afforded pure compound **10** as the major product, in 24% yield.

Benzylation of both the phenol hydroxy groups, followed by hydrolysis of the pivaloyl group and oxidation of the resulting primary alcohol to the aldehyde afforded intermediate **12** in 50% yield over the three steps. A Horner-Wadsworth-Emmons olefination was then carried out with posphonate **13** in the presence of *tert*-BuOK in DMF to afford protected *C*-glucosyl resveratrol **15**(E) isolated in 55% yield, which (*E*)-configuration is confirmed by the coupling constant of the olefinic proton at δ8.13 (Fig.S10 in SI). Further debenzylation of **15** with BCl_3_ at low temperature afforded the desired compound **6**, in 35% yield.

### Effects on membrane dipole potential

**Figure 2:**
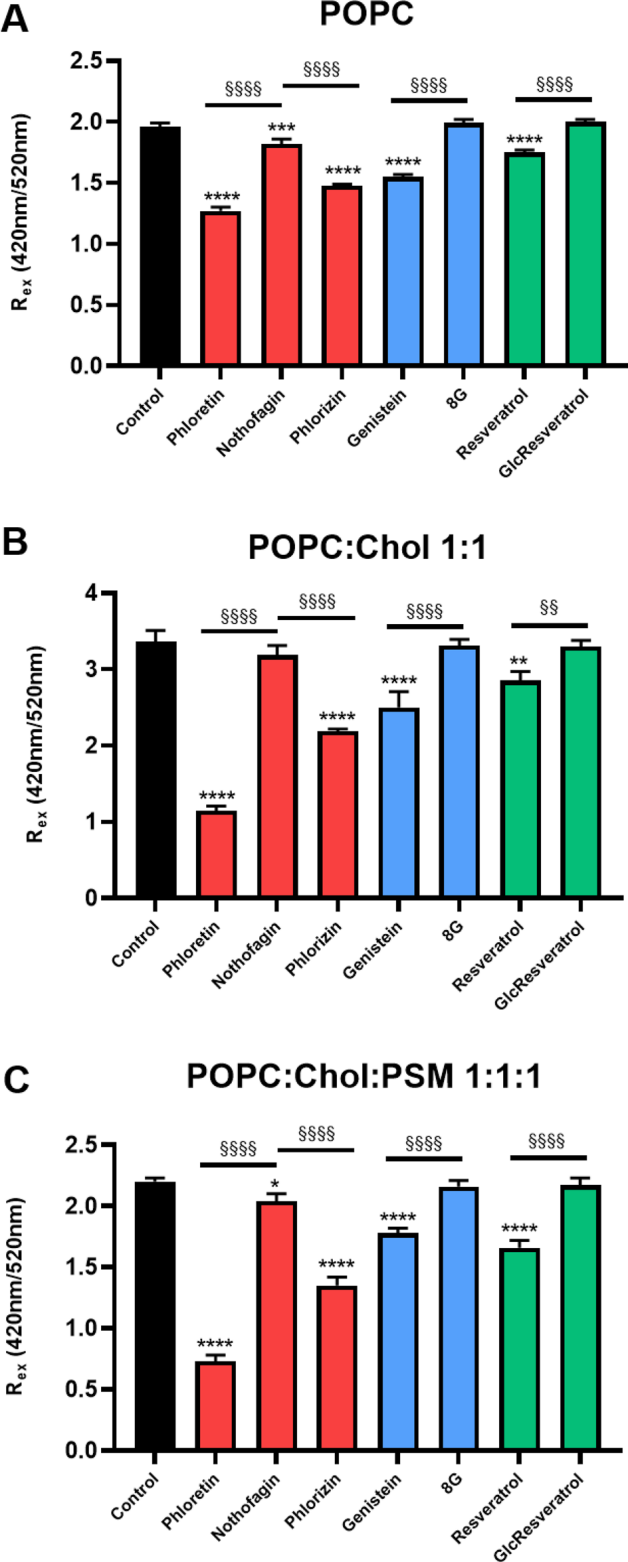
Membrane dipole potential measurements through the excitation intensity ratio at 420 nm / 520 nm of di-8-ANEPPS in pure POPC, POPC:Chol 1:1 and POPC:Chol:PSM 1:1:1 liposomes (1 mM total lipid) at 23 °C. Each compound was added to reach a final concentration of 50 μM. Probe: lipid ratio was 1: 500. Results are presented as the mean ± SD and each experiment was performed in triplicates. Statistical differences between compounds and control samples were assessed by one-way ANOVA followed by a Tukey’s post-test. **P < 0.01, ***P < 0.001, ****P < 0.0001 vs. control; ^§§^*P* < 0.01, ^§§§§^*P* < 0.0001 vs. aglycone. Chol - cholesterol;  8G  -8-Glucosylgenistein (**5**); GlcResveratrol- 4-Glucosylresveratrol (**6**).

Membrane dipole potential varies from *ca*. 200 to *ca*. 400 mV depending on the lipid composition of the bilayer, and was determined by di-8-ANEPPS fluorescence ratiometric measurements, *R*_ex_, between the intensity of the excitation spectra at 420 and 520 nm^43–45^. We used this approach to assess the impact of each polyphenol on membrane dipole potential of LUV with different lipid compositions, namely pure POPC, POPC:cholesterol 1:1 and POPC:cholesterol:PSM 1:1:1. Because di-8-ANEPPS can be a dipole modifier itself^46^, we used a very low molar ratio of probe: lipid (1:500), ensuring that probe is not influencing membrane properties, particularly the membrane dipole potential, as reported by Clarke et al^47^. These authors also showed that probe-probe interaction, probe aggregation or membrane saturation are negligible for this probe concentration. In accordance with the previously described and well-known ability of cholesterol to increase membrane dipole potential^44,48–51^, POPC:cholesterol LUV (*l*_o_ phase) presented significantly higher *R*_ex_ values than pure POPC liposomes (*l*_d_ phase), with a 1.5-fold increase, here displayed in Fig. 2. The inclusion of PSM in the chosen molar proportion, decreasing the mole fraction of cholesterol from 50 to 33%, which leads to *l*_d_ and *l*_o_ segregation in the ternary system, caused a decrease in this ratio to a value close to that observed for pure POPC LUV (2.20 ± 0.03 vs. 2.03 ± 0.02. The results are in overall agreement with our previous measurements in single, two and three component lipid mixtures using either di-8-ANEPPS or a similar probe, di-4-ANEPPS^35,50,52^.

Concerning the effect of the different compounds on membrane dipole potential, the absence of noticeable alterations of the spectral envelope in both excitation and emission spectra, and the absence of significant static quenching—the fluorescence intensity of the probe is not significantly altered by the addition of the compounds— support the absence of specific probe-compound interactions (Supplementary information, Fig. S2). Collisional quenching is also ruled out, as the fluorescence lifetime of the probe is either unchanged or slightly increased upon addition of the compounds (Supplementary Information, Table S1). In fact, the studies of the interactions of the polyphenol phloretin with di-8-ANEPPS *versus* those of the lipid 6-ketocholestanol with di-8-ANEPPS in ethanol solution show that hydrophobic interactions are dominant^47^, anticipating that in a membrane environment, where lipid is in large excess in relation to both probe and compound as in the present work, lipid-compound, and lipid-probe interactions predominate massively over probe-compound interactions. A complete absence of perturbation by the probe and of interactions between probe and compound is never attained, and these interactions maybe different for each compound. Thus, some authors have performed calibration of the effect of sterols and other dipole potential modifiers comparing probe-free methods with ratiometric measurements using di-8-ANEPPS^40,53,54^. We have also calibrated the changes in the ratiometric value of di-8-ANEPPS induced by several compounds studied in this work against the changes induced on membrane dipole potential, as measured through probe-independent approaches (see text under “Quantitative estimation of dipole potential alterations”). Ratiometric dyes have been extensively used to quantify dipole potential shifts due to changes in membrane composition, temperature, or the addition of multiple compounds with different structure and membrane interactions, and the effects are highly reproducible^43,44,49,50^. It was theoretically^55^ and experimentally^53^ demonstrated that the ratiometric method here used is not influenced by specific interactions. These results corroborate that the probe is solely responding to alterations in the membrane environment as consequence of compound incorporation and its effects on membrane properties.

In all lipid systems used herein, phloretin exerted the strongest decrease in the membrane dipole potential when compared to genistein and resveratrol. The observed decrease is in alignment with previous reports describing the dipole modifier-ability of phloretin and genistein^34,40,56–59^, being more dramatic (up to threefold) in POPC:Chol and POPC:Chol:PSM-containing LUV than in liposomes solely containing POPC.

This reflects a more pronounced effect in cholesterol-rich domains, which was also observed in the case of genistein and resveratrol. Indeed, the ability of these and other PAINS to promiscuously alter membrane protein function has been described and attributed to cell membrane perturbations rather than specific protein binding^17^. Given the role of membrane dipole potential in protein conformation and function, particularly in cholesterol-containing domains such as lipid rafts^60^, our results indicate that a decrease in the membrane dipole potential may be one of the reasons behind this type of PAINS behavior.

The molecular mechanism underpinning this effect is probably connected to the lipophilic character and/or structural planarity of these types of molecules, combined with the multiple hydroxy groups present in both ends of their structure–which are able to act as hydrogen bond donor and/or acceptor groups^35^. The fact that the only hydroxy group of cholesterol is protected by the polar headgroups of phospholipids to avoid contact with water, like an umbrella^60^, may allow these polyphenols to lay at a more superficial location in the membrane. Indeed, it is possible that they end up placed in the membrane in such a way that their dipole moment is aligned with that of the membrane in an anti-parallel manner, causing the observed reduction in the overall membrane dipole potential. Resveratrol, for instance, has recently been shown to be distributed more at the surface of saturated 1,2-dipalmitoyl-PC (DPPC) monolayers, promptly establishing hydrogen bonds with the charged phosphate oxygen atoms of the phospholipid headgroups and/or carbonyl oxygen atoms of the acyl moieties^61^. This superficial positioning in the membrane may further lead to a remodelling effect through the rearrangement of existing hydrogen-bond networks between phospholipid headgroups and surface water molecules, which mediate membrane hydration mechanisms. In cholesterol-containing and more complex lipid systems, these interactions may even block the binding sites of the hydroxy group of cholesterol in phospholipids and sphingolipids^62^ leading to alterations in membrane thickness and fluidity^63^. In accordance, quercetin–a paradigmatic example of polyphenols with diverse and even promiscuous bioactivities–was recently shown to increase membrane hydration by interfering with cholesterol/sphingolipid-enriched domains, and is, like resveratrol, more superficially located in complex lipid mixtures^64^.

Notably, the *C*-gluycosylated compounds show a completely different behavior. Our results clearly show that the observed dipole potential-decrease exerted by phloretin, genistein and resveratrol was fully abolished by the introduction of *C*-glucosyl moieties in each one of the three compounds (Fig. 2). The effects of nothofagin were the most dramatic ones when compared to those of its aglycone, phloretin, and even though the statistical comparison of *R*_ex_ by means of a One-Way ANOVA still finds significant differences between LUV containing nothofagin and the corresponding control in each lipid system, a broader Two-Way analysis including all liposome compositions points towards the inability of *C*-glucosides to change the membrane dipole potential (see Supplementary Information, Fig. S1).

In order to validate our experimental approach and to assess how specific could be the role of *C*-glucosylation, we also studied the effect of phlorizin, i.e., a *O*-glucosyl derivative of phloretin, on the membrane dipole potential in the three lipid mixtures. Phlorizin has been reported to be a dipole potential modifier with a behavior qualitatively similar to phloretin^65^. As can be seen in Fig. 2, our results confirm that phlorizin is, indeed, a dipole potential modifier, i.e., the *R*_ex_ parameter of di-8-ANEPPS decreases with the addition of phlorizin in all three lipid systems, although not as strong as the aglycone. These results further strengthen the importance of *C*-glucosylation for the abolishment of the dipole potential modifying ability of this polyphenolic compound.

Changes in the dipole moment of small molecules were recently shown to exert a significant impact on their location in the lipid bilayer^66^. Moreover, sugar hydroxy groups may compete with phenolic binding to phospholipid headgroups, further promoting a new orientation and placement of the aglycone within the membrane. On top of the higher ability of *C*-glucosides to establish hydrogen bonds with water molecules present at the membrane surface, the polyhydroxy nature of sugar moieties–which is very different from that of polyphenolic OH groups that are part of resonant aromatic rings–produces multiple small electric dipoles within the polar head of these *C*-glucosyl polyphenols, therefore inducing relevant changes in membrane hydration mechanisms. Indeed, POPC headgroups are endowed with a single large electric dipole inherent to a nitrogen-centered positive charge, which is, therefore, evenly oriented. This leads to a water-structuring effect that creates hydration-associated repulsion forces in the membrane^63^. In contrast, glycolipid membranes are able to counter-act such effects through the remodelling of hydrogen bonding patterns that modulate membrane surface hydration mechanisms, namely through lipid-lipid, lipid-water and water-water interactions. As a consequence, water molecules at the surface of the membrane are not subjected to such a strong alignment pressure, and repulsion due to water structuring becomes non-relevant in glycolipid membranes^67^. Lower repulsion allows a higher cohesion of water molecules at the membrane surface, affecting dramatically their influence on membrane dipole potential^68,69^.

Previous studies have shown that phloretin is able to permeate membranes^70^ but *C*-glucosylation may affect this behavior with possible influence on the extent of the membrane perturbations induced by the polyphenol. Recently, we have tested the effect of *C*-glucosylation on membrane permeation for the pair genistein and 8-glucosylgenistein and found no significant difference between their effective permeability^71^.

In light of our results, we propose that polyphenol *C-*glucosides mimic the water-remodelling effects observed in glycolipid membranes, leading to an increase in superficial water compactness and, thus, contributing to a larger membrane dipole potential when compared to their corresponding aglycones. Overall, the different effects proposed above for the influence of the *C*-glucosyl group cancel the decrease in dipole potential that would be due to the aglycone moiety alone.

At this point, it is important to highlight that *C*-glucosides could maintain some of the flavonoid interactions with relevant therapeutic targets, and in some cases could even enhance bioactivity, despite their incapacity to alter membrane dipole potential. Indeed, nothofagin (**4**) is actually a highly selective SGLT2 nanomolar inhibitor^38^ with antidiabetic, antithrombic and diuretic effects, being furthermore able to prevent high-glucose inflammation in vivo^69,72^. 8-Glucosylgenistein (**5**) is also a potent antidiabetic agent in vivo, with a strong inhibitory activity towards the formation of human islet amyloid polypeptides oligomers and fibrils^41^.

### Quantitative estimation of dipole potential alterations

In the work by Chulkov et al.^58^ the changes in the membrane dipole potential (Δ ψ_d_) induced by genistein, phloretin and phlorizin, measured by a non-fluorescence method in ternary 1,2-dioleoyl-PC (DOPC):Chol:SM (57:33:10 mol%) membranes were − 48 ± 10 mV, − 153 ± 18 mV and − 104 ± 5 mV, respectively. Thus, the effect of phloretin was quantitatively larger than that of phlorizin which was, in turn, larger than that of genistein. This is in full quantitative agreement with our measurements through di-8-ANEPPS ratiometric method.

These compounds were also studied by Efimova and Ostroumova^59^ for different membrane compositions. Using the values of Δψ_d_ retrieved from these works and our *R*_ex_ values, we found that there is a very good linear correlation between them (not shown). Therefore, our results can be used to quantitatively estimate dipole potential changes induced by the compounds. In addition, we have noticed that the variation in *R*_ex_ previously obtained by us for the mixtures POPC:cholesterol and POPC:ergosterol with di-4-ANEPPS^50^, a probe very similar to di-8-ANEPPS is parallel to the trend in dipole potential reported for the same systems by Haldar et al.^44^, and the trend observed by Starke-Peterkovic et al.^49^ with 1,2-dimyristoyl-PC (DMPC):cholesterol upon increasing sterol concentration at 30 °C. Finally, the variation in *R*_*ex*_ between POPC and POPC:cholesterol obtained by us with di-8-ANEPPS (Fig. 2) parallels the change in dipole potential measured by Gross et al.^53^ and Szabo^51^ between egg-PC and egg-PC:cholesterol (1:1 mol:mol) lipid bilayers by fluorescence independent methods.

In order to quantitatively estimate the changes in membrane dipole potential, we have taken the linear relationship between *R*_ex_ and ψ_d_ given by Starke-Peterkovic T. et al. (Eq. 1)^45^, and we have rescaled our data to account for the different wavelengths used in each work. Since we were interested only in obtaining dipole potential changes, the intercept, which was the parameter with larger relative error in the authors equation, does not interfere with our calculations.

1 $$\uppsi _{{\text{d}}} \left( {{\text{mV}}} \right) \, = \, (R_{{{\text{ex}}}} + \, (0.{3} \pm \, 0.{4})) \, / \, ({4}.{3} \pm { 1}.{2}) \times {1}0^{{ - {3}}}$$

Rescaling of our data was performed to match the dipole potential values reported by Haldar et al.^44^ in the POPC and POPC:cholesterol systems through application of Eq. (1). Instead of rescaling the data, an equivalent procedure is to recalculate the slope value and thereby it is possible to obtain the dipole potential changes induced by the compounds directly from our data in Fig. 2, through application of Eq. (2) (taking the difference betwween *R*_ex_ of the sample with compound and the control). This equation can be applied to di-8-ANEPPS ratiometric measurements when the excitation and emission wavelengths are the same as used in this work.

2 $$\Delta\uppsi _{{\text{d}}} \left( {{\text{mV}}} \right) = \, \left( {{365 } \pm { 1}0{2}} \right).\Delta R_{{{\text{ex}}}}$$

The calculations led to the Δψ_d_ values given in Table 1. The Δψ_d_ induced by phloretin on POPC LUVs is identical to the value reported for phloretin on egg-PC (major component: POPC) planar lipid membranes, measured by Hidaka and Asami^73^ by dielectric spectroscopy. The value we obtained for phloretin is also in quantitative agreement with the studies by Gross et al.^53^ and Cseh and Benz^74^.

**Table 1:** Membrane dipole potential changes (Δψ_d_, mV) induced by the compounds studied on LUV made of lipid mixtures with the compositions indicated, at 23 °C. The values were calculated using Eq. (2) and the error was obtained considering the experimental error in *R*_ex_ measurements and the uncertainty of the proportionality constant in Eq. (2). GlcResveratrol - 4-Glucosylresveratrol; 8G – 8-Glucosylgenistein.

Δψ_d_ (mV)	POPC	POPC:cholesterol	POPC:cholesterol:PSM
Phloretin	− 103 ± 34	− 327 ± 100	− 218 ± 69
Nothofagin	− 22 ± 12	− 25 ± 24	− 24 ± 16
Phlorizin	− 72 ± 22	− 173 ± 53	− 126 ± 45
Genistein	− 61 ± 19	− 127 ± 66	− 62 ± 23
8G	4 ± 6	− 7 ± 14	− 5 ± 9
Resveratrol	− 32 ± 12	− 76 ± 39	− 80 ± 31
GlcResveratrol	5 ± 5	− 8 ± 14	− 4 ± 9

Genistein is a weaker dipole modifying agent than phlorizin and its *O*-glucosyl derivative, genistin, is unable to change membrane dipole potential of ternary lipid mixtures (Δψ_d_ = − 1 mV)^58^. However, for phlorizin, only *C*-glucosylation affords a derivative completely lacking the ability to modify membrane dipole potential. Thus, our results suggest that *C*-glucosylation is a more powerful procedure to generate compounds counteracting the dipole potential modifying ability of polyphenols.

Considering our results in the ternary lipid mixture, which is closer to the composition of vertebrate cells plasma membrane outer leaflet, the decrease in dipole potential upon addition of polyphenols is 62 mV (genistein) or higher. Interestingly, a Δψ_d_ in the order of—60 mV could cause a loss of ca. 70% of plasma membrane Na^+^/K^+^-ATPase activity^45^. A drop of the dipole potential induced by lyotropic anions, such as perchlorate, inhibited the conformational transition E_1_P(Na^+^)_3_ → E_2_P(Na^+^)_3_ of the enzyme in its phosphorylated state^75^. In the same work, it was shown that a change of electrical potential of no more − 16 mV would be sufficient to produce the decrease of affinity for ATP binding experimentally determined by the authors. Therefore, even considering possible shielding effects that could limit the effect of dipole modifying agents in a biological context, the values of Δψ_d_ for the aglycones and phlorizin in Table 1 can have physiological relevance.

### Do the tested polyphenols affect global membrane order and compactness?

To exclude other possible effects caused by the three polyphenol aglycones and their corresponding *C*-glucosides that could result from severe membrane disorganization or disruption, DPH-PC steady-state (SS) fluorescence anisotropy measurements in LUV were carried out in the same lipid systems used for dipole potential evaluation. The DPH group of DPH-PC is buried into the membrane, as expected from its hydrophobic character, and oriented in a parallel manner to the phospholipid fatty acid acyl chains^76^. This orientation allows the zwitterionic head of DPH-PC to be located at the membrane surface without the loss of the deep insertion of DPH. As shown by Kaiser et al.^76^, its average membrane location is closer to the bilayer center when compared to its analogue TMA-DPH and, in fact, deeper than DPH itself. It has been regarded as a relevant way of measuring lipid order in both artificial and biological membranes and was, therefore, chosen to probe the effects of each compound in our experiments^77,78^.

**Figure 3:**
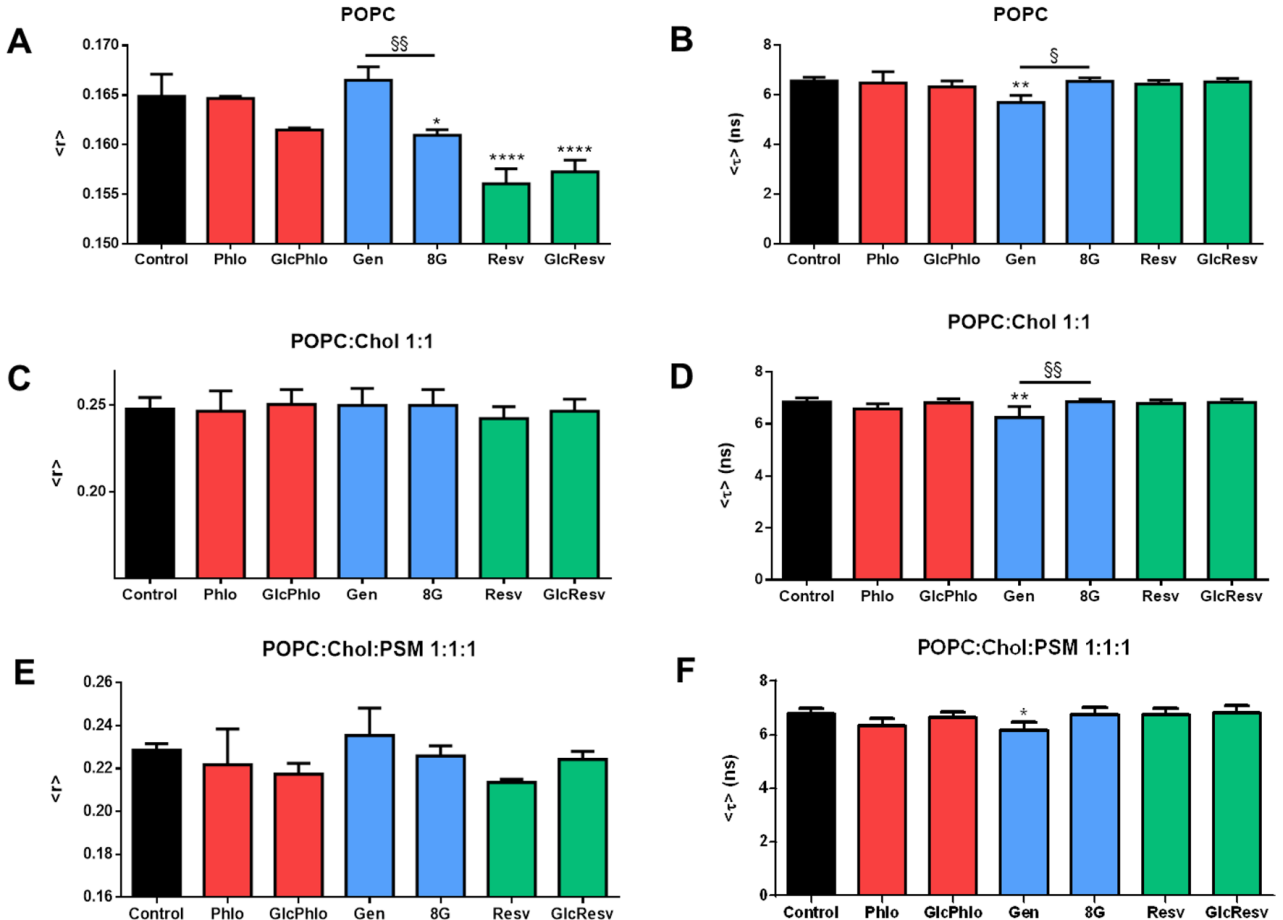
Fluorescence spectroscopy analysis of membrane properties at 23 °C in LUV of (**A**,**B**) pure POPC, (**C**,**D**) POPC:Chol 1:1 and (**E**,**F**) POPC:Chol:PSM 1:1:1 (1 mM total lipid) by means of DPH-PC steady-state anisotropy and fluorescence lifetime measurements, with a probe: lipid ratio of 1: 500. (**A**,**C**,**E**) DPH-PC steady-state fluorescence anisotropy; (**B**,**D**,**F**) DPH-PC mean fluorescence lifetime (fluorescence intensity decay analysis results fully available in Supplementary Information–Table S1). Each compound was added to reach a final concentration of 50 μM. Results are presented as the mean ± SD and each experiment was performed in triplicates. Statistical differences between compounds-containing and control samples were assessed by one-way ANOVA followed by a Tukey’s post-test. *P < 0.05, **P < 0.01 and ****P < 0.0001 vs. control; §*P* < 0.05 and §§*P* < 0.01 vs. aglycone. Chol - cholesterol; Phlo – phloretin (**1**); GlcPhlo – nothofagin (**4**); Gen – genistein (**2**); 8G – 8-glucosylgenistein (**5**); Resv – resveratrol (**3**); GlcResv – 4-glucosylresveratrol (**6**).

As expected from the previously described compacting effects of cholesterol^39,79^, the introduction of this sterol in the membrane significantly increases membrane order and packing, as manifested by the increase in DPH-PC SS fluorescence anisotropy in POPC:cholesterol liposomes when compared to pure POPC LUV, from 0.165 ± 0.002 to 0.247 ± 0.007 (Fig. 3A vs. Figure 3C; *P* < 0.0001). Segregation of *l*_d_ and *l*_o_ phases in the ternary lipid system then led to a slight but statistically significant increase in membrane fluidity (Fig. 3C vs. Figure 3E; *P* < 0.01), as expected for an *l*_d_ mole fraction of ca. 17%^39^ and an *l*_d_/*l*_o_ partition coefficient of the probe close to 1^76^.

The fluorescence intensity decays of DPH-PC could all be described by a bi-exponential function, with one component lifetime of ca. 3 ns and another of ca. 7 ns in agreement with previous studies^80,81^. Representative experimental decays of DPH-PC can be found in Fig. S3 in Supplementary Information and the fitting parameters averaged over the different replicates are given in Table S2. DPH-PC mean fluorescence lifetime was identical in the three different lipid systems, thus indicating that, in the regions where this probe is placed, membrane hydration and dielectric constant, which are known to alter the fluorescence intensity decay of DPH fluorophore^81–83^, are not markedly different for those membrane lipid compositions at 23 °C (Fig. 3B,D,F).

In polyphenol-containing samples, none of the compounds had a dramatic effect on the SS fluorescence anisotropy of DPH-PC. However, resveratrol and its *C*-glucosyl derivative, followed by 8-glucosylgenistein and, to a minor extent, nothofagin (with a significance of 0.06 in a Student’s t- test *vs.* control and < 0.001 *vs*. phloretin) were able to impact membrane packing in POPC liposomes (*l*_d_ phase), as revealed by the decrease in DPH-PC SS fluorescence anisotropy when compared to controls (Fig. 3A). This points towards slight membrane fluidification in the presence of the stilbenes and *C*-glucosyl polyphenols. However, the decrease was found to be non-significant in the *l*_o_ and *l*_d_ + *l*_o_ lipid systems (Fig. 3C,E, respectively), which are more relevant in the assessment of protein functioning disturbance. In contrast, the previously observed tendency of genistein to decrease membrane fluidity ^84,85^ was not detected in our experiments, as no significant changes in SS fluorescence anisotropy of the probe were observed in the presence of this compound. However, the fluorescence lifetime of DPH-PC was shorter in the presence of genistein in the three lipid mixtures studied (Fig. 3B,D, F), indicating that this molecule affects membrane polarity in the regions probed by DPH-PC.

The results here presented using DPH-PC as a reporter for membrane properties, showing small or no alterations, are consistent with our previous studies on rosmarinic and chlorogenic acids^35^. Although none of the two phenolic acids affected global membrane order nor hydration/polarity, these compounds were able to decrease membrane dipole potential in both *l*_d_ and *l*_o_ bilayers and elicit profound rearrangements of membrane lipid domains in *l*_d_/*l*_o_ mixtures.

It could be possible that a smaller effect of the *C*-glucosylated compound when compared to that of the aglycone in the dipole potential would result from a weaker membrane partition. However, the results of DPH-PC SS fluorescence anisotropy show that in POPC bilayers the *C*-glucosylated compounds are partitioning to the membranes, as they have an effect on membrane fluidity (Fig. 3A), in some cases surpassing the effect of the respective aglycone (phloretin and genistein). Both the aglycone and the glucosides are able to insert in the bilayer, although the aglycone has a deeper penetration. Such behavior has been observed in molecular dynamics simulations by Trouillas and coworkers for other polyphenol glycosides^86^. The sugar moiety forces the molecule to adopt a more superficial location in the membrane and establishes more hydrogen bonds with the hydration water and phospholipid headgroups. Hence, the *C*-glucosyl polyphenol can induce a perturbation that propagates deeper in the bilayer, as the lipid molecules are unable to pack so tightly, increasing bilayer fluidity as shown by the experiments with DPH-PC. A similar trend was found in an experimental study of the perturbation of liposomes induced by genistein and some genistein glycosides^87^. Moreover, we found that quercetin in POPC:cholesterol 1:1 bilayers, where it has superficial location, decreases the order parameters in POPC acyl chain carbons (C2–C9 in *sn*-1 and C2–C7 in *sn*-2)^64^. In addition, the polyphenol adopts a different orientation in the membrane when it is linked to a sugar^86,88^, which may also help to explain why 8-glucosylgenistein influences more membrane fluidity than its aglycone, genistein. On the other hand, the distinct location and orientation of the molecule may also explain the decreased ability of 8-glucosylgenistein to modify the membrane dipole potential. In the case of resveratrol and *C*-glucosyl resveratrol, both compounds have the most significant effect on membrane fluidity. However, the effect on membrane dipole potential is apparent only for the aglycone. Thus, even *C*-glucosylated compounds with the ability to increase membrane fluidity, and therefore unequivocally partitioning to the membrane phase, do not behave as dipole potential modifiers, in contrast to their aglycone counterpart.

## Conclusion

**Figure 4:**
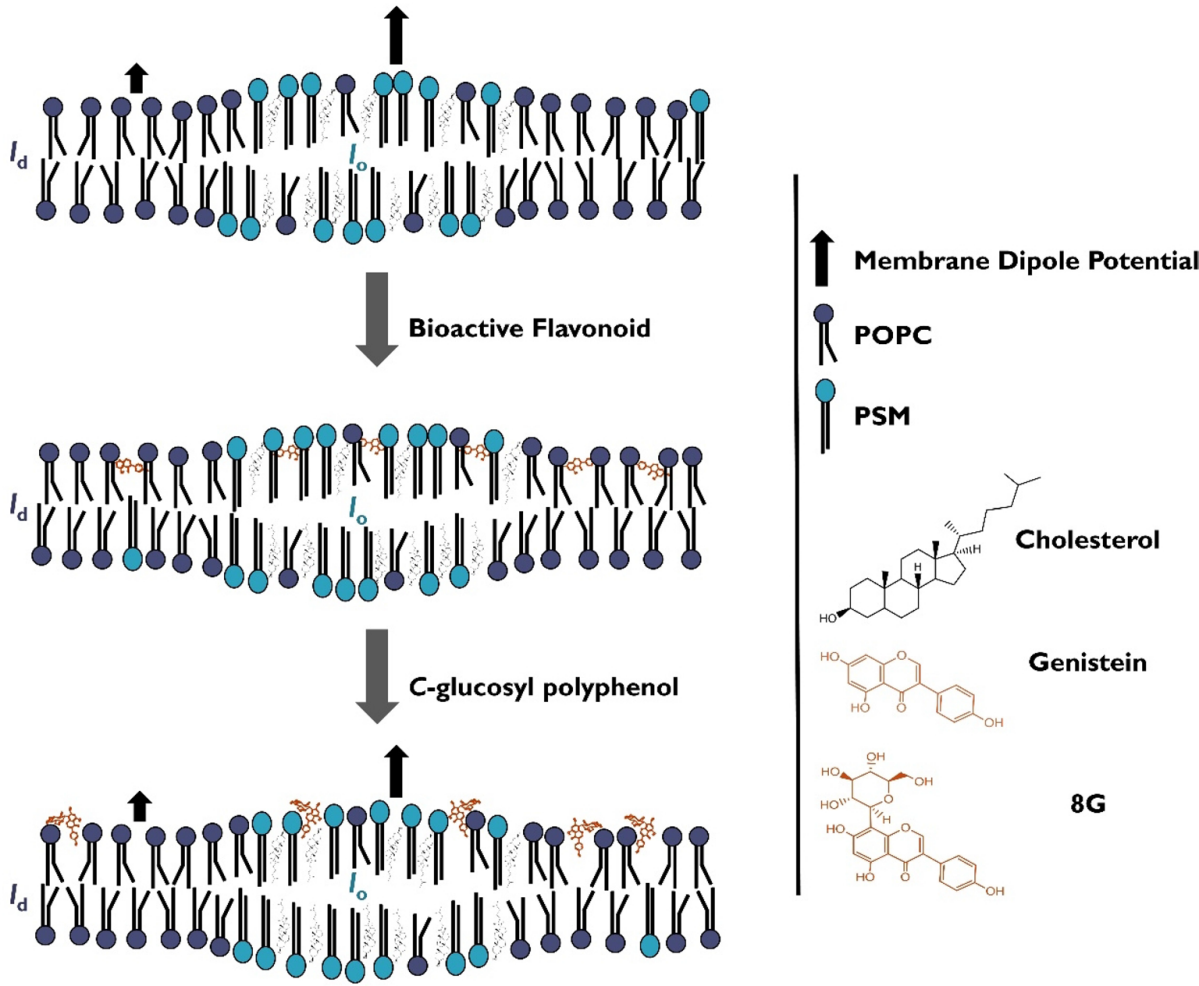
PAINS-like polyphenols and their *C*-glucosides affect differently the membrane dipole potential. This schematic illustration represents a pictorial view of a ternary lipid mixture with *l*_d_/*l*_o_ phase coexistent, in the absence (top) and in the presence of genistein (middle) or 8-glucosylgenistein (8G) (bottom). These compounds are placed in the membrane with the polyphenol at different depth and orientation, inspired in previously reported theoretical and experimental work^89–92^. The *C*-glucosyl polyphenol has, indeed, a more superficial location due to the sugar moiety. Together with the H-bonding pattern of the sugar, the location and the orientation of the *C*-glucosyl polyphenol contribute to its performance as a non-dipole modifier.

We have studied a set of polyphenols with known PAINS-type behavior related to membrane perturbation or membrane dipole modifying activity. After synthesizing their *C*-glucosyl derivatives, we assessed whether the interaction with complex membrane models would be altered in the presence of the sugar moiety. Notably, the first synthesis of 4-glucosylresveratrol (**6**) was accomplished for this purpose, and this expedient route is herein presented. Our DPH-PC SS fluorescence anisotropy results do not support the hypothesis that aglycones **1–3** owe their membrane-interfering behavior to the induction of changes in membrane order and compactness, at least at the level of the membrane hydrophobic core. However, we do have consistent results allowing to deduce that they are related to a membrane dipole modifying activity, as shown by di-8-ANEPPS fluorescence spectroscopy ratiometric measurements, with greater differences observed for cholesterol-containing membranes when compared to controls. These results indicate that PAINS may interfere with cholesterol-rich domains such as lipid rafts, probably by affecting the interactions at the membrane/water interface, with potential consequences in terms of the regulation of protein conformation and activity, lipid and protein sorting and trafficking, and signal transduction. Furthermore, all *C*-glucosides were able to fully prevent these changes in membrane dipole potential, as the membrane dipole potential observed in their presence did not differ from that of normal controls (LUV in the absence of any compound). We suggest that the sugar moiety alters the capacity to rearrange hydrogen-bond networks and hydration layer at the membrane surface, most likely due to the creation of multiple small electric dipoles by their hydroxy groups, which may therefore counteract the dramatic changes in the membrane dipole potential caused by the aglycones. This hypothesis is here exemplified for the pair genistein (**2**) / 8-glucosylgenistein (**5**) in Fig. 4. Given the reported strong impact of these changes in membrane function, including transmembrane protein conformation and activity, this study may open new doors for the investigation of natural products with known biological activities without the risk of generating false positive results associated with membrane disruption effects, which directly relate to PAINS-type behavior.

## Methods

### Chemical synthesis

HPLC grade solvents and reagents were obtained from commercial suppliers and were used without further purification. The syntheses of phloretin (**1**), nothofagin (**4**) and 8-glucosylgenistein (**5**) were conducted according to previously described methodologies^38,41^. Genistein (**2**) and resveratrol (**3**) were purchased from Sigma Aldrich and TCI chemicals, respectively. All reactions were monitored by thin layer chromatography (TLC), which was carried out on aluminum sheets (20 × 20 cm) coated with silica gel 60 F-254, 0.2 mm thick (Merck) with detection by charring with 10% H_2_SO_4_ in ethanol. Column chromatography (CC) was performed using silica gel 230–400 mesh (Merck). Melting points were measured with a SMP3 melting point apparatus, Stuart Scientific, Bibby. Optical rotations were measured with a PerkinElmer 343 polarimeter. Nuclear Magnetic Resonance (NMR) experiments were recorded on a Bruker Avance 400 spectrometer at 298 K, operating at 100.62 MHz for ^13^C and at 400.13 MHz for ^1^H for solutions in CDCl_3_, (CD_3_)_2_CO or MeOD (Sigma-Aldrich). Chemical shifts are expressed in δ (ppm) and the proton coupling constants *J* in Hertz (Hz), and spectra were assigned using appropriate COSY, DEPT, HMQC, and HMBC spectra. Carbon labeling for NMR assignment was as follows: The olefinic carbons are labelled as C-a and C-b, ring A is labelled from C-1 to C-6, ring B from C-1′ to C-6′ and glucosyl carbons as C-1ʺ to C-6ʺ. The abbreviation Piv is used for the group COC(CH_3_)_3_ . High resolution mass spectra of new compounds were acquired on a Bruker Daltonics HR QqTOF Impact II mass spectrometer (Billerica, MA, USA). The nebulizer gas (N_2_) pressure was set to 1.4 bar, and the drying gas (N_2_) flow rate was set to 4.0 L/minute at a temperature of 200 °C. The capillary voltage was set to 4500 V and the charging voltage was set to 2000 V.

### Synthesis of 3,5-dihydroxybenzyl 2,2-dimethylpropanoate (**9**)

 Pivaloyl chloride (516 μL, 4.19 mmol) was added in 3 portions to a solution of 5-(hydroxymethyl)benzene-1,3-diol **8** (0.50 g, 3.5 mmol, 0.8 eq.) and DMAP (0.085 g, 0.69 mmol, 0.16 eq.) in pyridine (8 mL) at 0 °C. The mixture was stirred at room temperature. After 18 h another portion of pivaloyl chloride (86 μL, 0.7 mmol, 0.17 eq.) was added and the mixture stirred for another 2 h. The crude was washed with HCl 2 M and extracted with DCM. The residue was dried with MgSO_4_ and the solvent evaporated under reduced pressure. The residue was purified by column chromatography (CyHex/EtOAc 10:1 → 6:1) to render compound **9** in 60% yield. R_*f*_ (CyHex/EtOAc 1:1) = 0.64; ^1^H NMR (CDCl_3_) δ (ppm) 6.38 (s, 2H, H-2, H-6), 6.30 (s, 1H, H-4), 4.97 (s, 2H, C*H*_2_-OPiv), 1.20 (s, 9H, C*H*_3_). ^13^C NMR (CDCl_3_) δ (ppm) 179.6 (CO), 157.1 (C-3 and C-5), 139.1 (C-1), 107.2 (C-2 and C-6), 102.7 (C-4), 66.1 (*C*H_2_), 39.0 (OCO*C*q), 27.3 (*C*H_3_). HRMS-ESI (*m/z*): [M + H]^+^ calcd for C_12_H_16_O_4_ 225.1121, found 225.1122.

### Synthesis of 3,5-dihydroxy-2-(2,3,4,6-tetra-*O*-benzyl-β-d-glucopyranosyl)benzyl 2,2-dimethylpropanoate (**10**) and 3,5-dihydroxy-4-(2,3,4,6-tetra-*O*-benzyl-β-d-glucopyranosyl)benzyl 2,2-dimethylpropanoate (**11**)

 Glycosyl donor **7** (6.20, 11.5 mmol) and compound **9** (3.6 g, 16.1 mmol, 1.4 eq.) were dissolved in a 1:1 mixture of DCM/ACN (80 mL). Drierite (500 mg) was then added, and the reaction was kept at –40 °C under N_2_ atmosphere. TMSOTf (4.2 mL, 23 mmol, 2 eq.) was added at low temperature and then the reaction stirred overnight at room temperature. After adding few drops of NEt_3_, the crude was washed with NaHCO_3_, extracted with DCM, washed with brine, dried over MgSO_4_ and concentrated in vacuum. The residue was purified by column chromatography (P.Ether/Acetone 10:1 → 7:1), affording a mixture of regioisomers **10** and **11** in 43% global yield. Two more column chromatography purification steps were necessary to isolate compounds **10** and **11** in 24% and 19% yield, respectively.

### 3,5-dihydroxy-2-(2,3,4,6-tetra-*O*-benzyl-β-d-glucopyranosyl)benzyl 2,2-dimethylpropanoate (**10**)

 R_*f*_ (P.Ether/Acetone 3:1) = 0.44; ${\upalpha }_{{\text{D}}}^{20}$ =  + 40° (*c* 1, CHCl_3_); ^1^H NMR (CDCl_3_) δ 7.33–7.26 (m, 12H, PhC*H*), 7.23–7.10 (m, 6H, PhC*H*), 6.94 (dd, *J* = 7.5 Hz, 1.6 Hz, 2H, Ph-Bn), 6.45 (d, *J*_*4,6*_ = 2.4 Hz, 1H, H-6), 6.42 (d, *J*_*4,6*_ = 2.4 Hz, 1H, H-4), 5.23, 5.20 (part A of AB system, *J* = 12.9 Hz, 1H, C*H*_2_OPiv), 4.97, 4.94 (part A of AB system *J* = 11.6 Hz, 1H, PhC*H*_2_-4ʹ), 4.90–4.84 (m, 3H, part B of AB system,C*H*_2._OPiv, part B of AB system,PhC*H*_2_-4ʹ, part A of AB system, PhC*H*_2_-3ʹ), 4.70 (d, *J* = 9.6 Hz, 1H, H-1ʹ), 4.60, 4.57 (part A of AB system, *J* = 12.3 Hz, 1H, PhC*H*_2_-6ʹ), 4.52, 4.49 (Part B of AB system, J = 10.5 Hz, PhC*H*_2_-3ʹ); 4.45, 4.42 (Part B of AB system, *J* = 12.3 Hz, PhC*H*_2_-6ʹ; part A of AB system, *J* = 12.3 Hz, 2H, PhC*H*_2_-2ʹ), 3.91 (t, *J* = 9.4 Hz, 1H, H-4ʹ), 3.86–3.64 (m, 5H, part B of AB system,PhC*H*_2_-2ʹ, H-2ʹ, H-3ʹ, H-6ʹa, H-6ʹb), 3.54 (d, *J* = 9.7 Hz, 1H, H-5ʹ), 1.18 (s, 9H, CH_3_). ^13^C NMR (CDCl_3_) δ 178.4 (CO), 157.9 (C-3), 157.0 (C-5), 138.6, 137.9, 137.7, 137.4 (Ph*Cq*), 136.6 (C-1), 128.5–127.6 (Ph*C*H), 113.8 (C-2), 108.4 (C-6), 104.9 (C-4), 86.2 (C-3ʹ), 81.4 (C-2ʹ), 78.6 (C-5ʹ), 77.1 (C-1ʹ, C-4ʹ), 75.7 (Ph*C*H_2_), 75.4 (Ph*C*H_2_), 75.3 (Ph*C*H_2_-4′), 73.4 (Ph*C*H_2_), 67.7 (C-6ʹ), 64.5 (*C*H_2_-OPiv), 38.8 [*C*(CH_3_)_3_], 27.2 (*C*H_3_). HRMS-ESI (*m/z*): [M + H]^+^ calcd for C_46_H_51_O_9_ 747.3528, found 747.3528.

### 3,5-dihydroxy-4-(2,3,4,6-tetra-*O*-benzyl-β-d-glucopyranosyl)benzyl 2,2-dimethylpropanoate (**11**)

 R_*f*_ (P.Ether/Acetone 3:1) = 0.39; ${\upalpha }_{{\text{D}}}^{20}$ =  + 18° (*c* 1, CHCl_3_). ^1^H NMR (CDCl_3_) δ 7.40–7.19 (m, 16H, PhC*H*), 7.15 (dd, *J* = 7.2, 1.9 Hz, 2H, PhC*H* ), 7.05–6.95 (m, 2H, PhC*H*), 6.48 (s, 2H, H-2, H-6), 5.01 (s, 2H, C*H*_2_-OPiv), 4.98 (s, 2H, PhC*H*_2_-3ʹ), 4.88 (d, *J* = 9.5 Hz 1H, H-1ʹ), 4.85, 4.82 (part A of AB system, *J* = 10.8 Hz, 1H, PhC*H*_2_-4ʹ ), 4.69, 4.66 (part A of AB system, *J* = 10.2 Hz, 1H, PhC*H*_2_-2ʹ), 4.61, 4.58 (part A of AB system, *J* = 12.0 Hz, 1H, PhC*H*_2_-6ʹ), 4.55, 4.52 (part B of AB system, *J* = 10.8 Hz, 1H, PhC*H*_2_-4ʹ), 4.47, 4.44 (part B of AB system, *J* = 12.0 Hz, 1H, PhC*H*_2_-6ʹ), 4.24, 4.21 (part B of AB system, *J* = 10.2 Hz, 1H, PhC*H*_2_-2′), 3.92 (t, 1H, H-4ʹ), 3.85–3.74 (m, 2H, H-3ʹ, H-2ʹ,H-6ʹa), 3.69, 3,67 (part BX of ABX system, *J*_*6′a,6′b*_ = 10.3, *J*_*5,6′b*_ 1.9 Hz, 1H, H-6ʹb), 3.58 (brd, 1H, , *J*_*4′,5′*_ = 9.6 Hz ,H-5ʹ), 1.24 (s, 9H, CH_3_). ^13^C NMR (CDCl_3_) δ 178.4 (CO), 155.6 (C3, C5), 138.7 (Ph*C´*), 138.3 (Ph*C*), 137.8, 137.6, 136.2 (Ph*C´*), 128.8–127.5 (Ph*C*H) 110.8 (C-4), 109.1 (C-2, C-6), 86.3 (C-3ʹ), 82.3 (C-2ʹ), 78.7 (C-5ʹ), 77.1 (C-4ʹ), 76.3 (Ph*C*H_2_-2ʹ), 75.6 (C-1ʹ), 75.3 (Ph*C*H_2_-4ʹ), 75.3 (Ph*C*H_2_-4ʹ), 73.4 (Ph*C*H_2_-6ʹ), 67.4 (C-6ʹ), 65.5 (*C*H_2_-OPiv), 38.8 [*C*(CH_3_)], 27.3 (*C*H_3_). HRMS-ESI (*m/z*): [M + H]^+^ calcd for C_46_H_51_O_9_ 747.3528, found 747.3526.

### Synthesis of 3,5-dihydroxy-2-(2,3,4,6-tetra-*O*-benzyl-β-d-glucopyranosyl)benzaldehyde (**12**)

*C*-glucosyl phenol **10** (1.52 g, 2.03 mmol) was dissolved in DMF (10 mL). To this solution, potassium carbonate (0.78 g, 5.68 mmol, 2.8 eq.) was added at 0 °C and stirred for 10 min at 0 °C. Then, benzyl bromide (0.67 mL, 5.68 mmol, 2.8 eq.) was added and the reaction was stirred at room temperature for 1.5 h. After completion, the reaction was neutralized with HCl, 2 M, extracted with DCM, washed with brine, dried over MgSO_4_, and concentrated under vacuum. The benzylation product was isolated by column chromatography (P.Ether/Acetone 11:1 → 9:1) in 85% yield. Lithium hydroxide dissolved in a 1:1 mixture of MeOH/H_2_O (24 mL) was added to a solution of the *C*-glycosyl compound (0.70 g, 0.76 mmol) in MeOH (10 mL). The mixture was refluxed for 36 h and then neutralized with Amberlite IR 120 H^+^. After filtration and evaporation of the solvent, purification was carried out by column chromatography (Hex/EtOAc 5:1 → 4:1) to render the hydrolysed product in 68% yield. Finally, to a suspension of pyridomium chlorochromate (0.33 g, 1.53 mmol) in DCM (8 mL), a solution of the alcohol in DCM (8 mL) was added. The reaction was stirred at room temperature for 3 h and, after completion, the residue was washed with H_2_O and extracted with DCM. The product was purified by column chromatography (Hex/EtOAc 6:1 → 5:1) to afford compound **12** in 81% yield. R_*f*_ (Hex/EtOAc 5:1) = 0.35; ${\upalpha }_{{\text{D}}}^{20}$ = -7° (*c* 1, CHCl_3_); ^1^H NMR (CDCl_3_) δ 10.76 (s, 1H, C*H*O), 7.47–7.06 (m, 29H, PhC*H*, H-6), 6.88 (brd, *J* = 6.8 Hz, 2H, PhC*H*), 6.74 (d, *J* = 2.4 Hz, 1H, H-4), 5.25 (d, *J* = 9.8 Hz, 1H, H-1ʹ), 5.09 (s, 2H, PhC*H*_2_-phenol), 4.96–4.81 (m, 5H, PhC*H*_2_-phenol, 2xPhC*H*_2,_ ), 4.62–4.42 (m, 4H, PhC*H*_2_), 4.02, 3,99 (Part B of AB system, *J* = 11.0 Hz, 1H, PhC*H*_2_), 3.85–3.64 (m, 5H, H-3ʹ, H-4ʹ, H-2ʹ, H-6a´, H-6ʹb), 3.59 (brd, *J* = 8.4 Hz, 1H, H-5ʹ). ^13^C NMR (CDCl_3_) δ 192.2 (CO), 159.3 (C-5), 158.1 (C-3), 138.5, 138.06, 138.04, 137.7 (Ph*C*), 137.5 (C-1), 136.4, 136.2, (P*C*), 128.6—127.0 (Ph*C*H), 106.3 (C-4), 104.3 (C-6), 87.2 (C-3ʹ) , 79.6 (C-5ʹ), 78.2 (C-4ʹ, C-2ʹ), 76.3 (Ph*C*H_2_-2ʹ), 75.6 (Ph*C*H_2_-3ʹ), 74.5 (Ph*C*H_2_), 73.6 (C-1ʹ), 73.4 (Ph*C*H_2_-6ʹ), 71.1 (Ph*C*H_2-Phenol_), 70.2 (Ph*C*H_2-phenol_), 68.8 (C-6ʹ). HRMS-ESI (*m/z*): [M + H]^+^ calcd for C_55_H_53_O_8_ 841.3735, found 841.3730.

### Synthesis of (*E*)-5-(4-benzyloxystyryl)-1,3-bis(benzyloxy)-4-(2,3,4,6-tetra-*O*-benzyl-β-d-glucopyranosyl)benzene (**15**)

 Potassium *tert*-butoxide was added to a solution of benzaldehyde **12** (0.4 g, 0.47 mmol) and phosphonate **13** (0.16 g, 0.47 mmol, 1 eq.) in DMF at 0 °C. The mixture was warmed to room temperature and stirred for 2 h. After completion, the mixture was diluted with water, extracted several times with EtOAc, washed with brine, dried over MgSO_4_, and concentrated under vacuum. The residue was purified by column chromatography (P.Ether/Acetone 22:1 → 20:1) to afford a mixture of isomers *Z* and *E* (1:2.3 ratio) in 60% global yield. Further purification steps afforded compound **15** in 55% yield. R_*f*_ (Hex/Acetone 7:1)** = **0.52; ^1^H NMR (CDCl_3_) δ 8.13 (d, *J* = 16.2 Hz, 1H, H-a), 7.46–7.16 (m, 35H, PhC*H*), 6.98–6.89 (m, 3H, PhC*H*, H-6), 6.85–6.75 (m, 3H, PhC*H*, H-b ), 6.54 (s, 1H, H-4), 5.28 (d, *J* = 9.8 Hz, 1H, H-1ʹ´), 5.12 (s, 2H, PhC*H*_2_-phenol at C-5), 5.06 (s, 2H, PhC*H*_2_-phenol at C-4ʹ), 5.02–4.82 (m, 5H, PhC*H*_2_-3, PhC*H*_2_-3ʹ´, 1xPhC*H*_2_-4ʹ´), 4.74,4.71 (part A of AB system, *J* = 11.4 Hz, PhC*H*_2_-6ʹ´),4.68, 4.65 (part B of AB system, *J* = 11.4 Hz , PhC*H*_2_ -4ʹ´), 4.57, 4.54 (part B of AB system, *J* = 11.4 Hz, 1H, PhC*H*_2_ -6ʹ´), 4.43, 4.40 (part A of AB system, *J* = 10.8 Hz, 1H, 1xPhC*H*_2_ -2ʹ´), 3.96–3.85 (m, 4H, 1xPhC*H*_2_-2ʹ´, H-2ʹ´, H-4ʹ´, H-6a´´), 3.83–3.75 (m, 2H, , H-6b´´, H-3ʹ´), 3.60 (brd, *J* = 9.4 Hz, 1H, H-5ʹ´). ^13^C NMR (CDCl_3_) δ 159.3 (C-5), 158.6 (C-3), 158.4 (C-4ʹ), 140.5 (C-1), 138.7, 138.5, 138.4, 138.4 (Ph*Csugar*), 137.2, 136.9 136.9 (Ph*C-resveratrol*), 130.7 (C-1ʹ), 130.2 (C-b), 128.6—127.2 (37xPh*C*H), 126.0 (C-a), 117.8 (C-2), 115.1 (2xPh*C*H), 105.1 (C-6), 100.3 (C-4), 87.3 (C-3ʹ´), 81.9 (C-2ʹ´), 78.9 (C-5ʹ´), 77.7 (C-4ʹ´), 75.8 (Ph*C*H_2_), 75.1 (Ph*C*H_2_-4ʹ´), 74.2 (Ph*C*H_2_-2ʹ´), 74.1 (C-1ʹ´), 73.7 (Ph*C*H_2_-6ʹ´), 71.2 (Ph*C*H_2-_phenol at C-3), 70.1, 70.0 (, PhC*H*_2_-phenol at C-5, Ph*C*H_2_-phenol at C-4ʹ), 68.9 (C-6ʹ´). HRMS-ESI (*m/z*): [M + H]^+^ calcd for C_69_H_65_O_8_ 1021.4674, found 1021.4673.

### Synthesis of (*E*)-4-(β-d-glucopyranosyl)-5-(4-hydroxystyryl)benzene-1,3-diol(**6**)

 To a solution of compound **15** (0.10 g, 0.098 mmol) in DCM, BCl_3_ was added in a dropwise manner at -78 °C in N_2_ atmosphere. After stirring for 2 h, MeOH was added and then the solvent eliminated under reduced pressure in a 25 °C bath. The residue was purified by reverse phase column chromatography (H_2_O/MeOH 9:1 → 7:3) to give compound **6** in 35% yield. **R**_***f***_ (DCM/MeOH 3:1) = 0.42; ^1^H NMR (MeOD) δ 7.38 (d, *J*_ortho_ = 8.30 Hz, 2H, H-2′ and H-6′), 6.83–6.73 (m, 4H, H-3′, H-5′, H-a, H-b), 6.57 (s, 1H, H-6), 6.26 (d, *J*_meta_ = 1.4 Hz, 1H, H-4), 3.94–3.85 (m, 2H, H-1ʺ and part AX of ABX system H-6ʺ a), 3.81, 3.78 (part BX of ABX system, 1H, *J*_6_ʺ _b-6′'a_ = 12.04 Hz, *J*_6ʺ__b-5_ = 4.60 Hz, H-6ʺ b), 3.57–3.45 (m, 3H, H-2ʺ, H-3ʺ and H-4ʺ), 3.44–3.38 (m, 1H, H-5ʺ, overlapped with solvent signal). ^13^C NMR (MeOD) δ 159.0, 158.8, 158.5 (C-3, C-5, C-4′), 141.9 (C-1), 132.0 (C-5), 131.1 (C-b), 129.8 (C-a), 129.1 (C-2′ and C-6′), 116.6 (C-3′ and C-5′), 115.9 (C-2), 106.4 (C-6) 103.9 (C-4), 82.3 (C-5ʺ), 80.2 (C-2ʺ), 73.9 (C-1ʺ), 71.8, 71.4 (C-3ʺ, C-4ʺ), 62.8 (C-6ʺ). HRMS-ESI (*m/z*): [M + H]^+^ calcd for C_20_H_23_O_8_ 391.1387, found 391.1389.

### Fluorescence spectroscopy

POPC and PSM were obtained from Avanti Polar Lipids. Di-8-ANEPPS was obtained from Biotium. DPH-PC was purchased from Molecular Probes. Cholesterol, minimum 99% and all other reagents, analytical grade were obtained from Sigma-Aldrich. Buffer solutions were prepared with ultrapure Milli-Q water at 18.2 MΩ.cm.

#### Lipid and probe quantification procedures

Phospholipid concentrations of POPC and PSM were determined gravimetrically and by inorganic phosphate quantification^93^. Chol quantification was made by gravimetry. Probe concentration in stock solutions was determined spectrophotometrically.

#### Preparation of large unilamellar vesicles (LUVs)

The interaction of plant polyphenols with lipid bilayers was studied by fluorescence spectroscopy in LUV suspensions, which were prepared according to previously described methods^94^. Briefly, the stock solution volume for the required final total lipid concentration was added to a vial, and the solvent evaporated with a mild, continuous flow of nitrogen, followed by overnight vacuum. Two identical samples were always prepared, with and without fluorophore, the latter to be used as blank. After hydration with buffer 10 mM HEPES (2-[4-(2-hydroxyethyl)piperazin-1-yl]ethanesulfonic acid), pH 7.4, 150 mM NaCl, samples were submitted to vortex stirring and freeze-thaw cycles. LUV suspensions were formed by extrusion (Avanti Mini-extruder) at 60 °C, by forcing the multilamellar vesicle suspension 21 times through polycarbonate filters with 100 nm diameter pores (Nuclepore, Whatman) and left to reach equilibrium overnight. The probes were added from stock solutions in ethanol to an aliquot of freshly prepared LUVs and equilibrated overnight to ensure complete probe incorporation into the membrane^49,95^. The same volume of solvent (less than 1% v/v) was added the other aliquot of LUV suspension, to be used as blank.

#### Fluorescence spectroscopy measurements

Fluorescence measurements were performed at 23 °C using a Horiba-Jobin Yvon Fluorolog 3–22 spectrofluorometer. The effect of compounds **1—6** on membrane fluidity and dipole potential was studied in POPC, POPC:Chol (1:1) and POPC:Chol:PSM (1:1:1) lipid bilayers labelled with DPH-PC (in MeOH) or di-8-ANEPPS (in EtOH), respectively. Probe/lipid ratio was 1:500 in all experiments (final lipid concentration was 1 mM and final probe concentration 2 μM). Compounds were dissolved in DMSO and added to the LUV suspensions to achieve a concentration of 50 μM, with a final DMSO concentration of 2%, followed by an incubation period of 2 h at 23 °C prior to fluorescence measurements. Steady-state fluorescence anisotropy, ‹*r*›, was calculated according to the following equation:

3 $$\it \left\langle r \right\rangle = \frac{{\left( {{\varvec{I}}_{{{\mathbf{VV}}}} - \user2{ GI}_{{{\mathbf{VH}}}} } \right)}}{{\left( {{\varvec{I}}_{{{\mathbf{VV}}}} + 2{\varvec{GI}}_{{{\mathbf{VH}}}} } \right)}}$$

in which *I*_XY_ represents the emission intensity reading with vertical (V) or horizontal (H) orientations of the excitation (X) and emission (Y) polarizers, and where *G*, obtained from the ratio of the intensities *I*_HV_/*I*_HH_, is a correction factor for the different detector sensitivity to vertical and horizontal polarized light. An adequate blank was subtracted from each intensity reading, and each set of four intensity components for each sample was measured seven times. Fluorescence anisotropy of DPH-PC was measured with λ_ex_ at 369 nm and λ_em_ at 450 nm, with a 2 nm bandwidth. The membrane dipole potential was calculated from the fluorescence intensity ratio obtained for di-8-ANEPPS at 420 nm and 520 nm excitation wavelengths after background correction using a blank (unlabelled) sample. The blank signal was always below 0.7% of the respective labelled sample. The excitation spectra were obtained from 400 to 625 nm with emission at 635 nm. None of the compounds absorb light in the region of the excitation band of di-8-ANEPPS used. Emission spectra were recorded between 470 e 650 nm, at an excitation wavelength of 460 nm. The bandwidth was 5 nm in both excitation and emission.

For time-resolved measurements by the single-photon-timing technique (SPT), nanoLED N-370 was used for the excitation of DPH-PC, and emission wavelength was 450 nm. The resolution of the detection system was 50 ps, and the number of counts in the peak channel was 10 000 − 20 000 for each sample. The time scale used for the analysis was 0.055517 ns/channel. The bandwidth was adjusted from the maximum value allowed by the instrumental setup (2.0–3.5 nm) to ensure an SPT regime. Data analysis was performed through a nonlinear least-squares iterative reconvolution method based on the Marquardt algorithm using the Time-Resolved Fluorescence Anisotropy Data Processor 1.4 program to obtain the fitting parameters. The fluorescence intensity decays were described by a sum of exponentials.

Considering that to each component i of the decay corresponds a normalized pre-exponential factor (amplitude) $\alpha_{i}$ and a lifetime $\tau_{i}$, the decay law can be given by

4 $$I\left( t \right) = \mathop \sum \limits_{i = 1}^{n} \alpha_{i} exp\left( { - \frac{t}{{\tau_{i} }}} \right)$$

The (intensity-weighted) mean fluorescence lifetime is then given by

5 $$<\tau> = \frac{{\sum \alpha_{i} \tau_{i}^{2} }}{{\sum \alpha_{i} \tau_{i}^{ } }}$$

### Statistical analysis

Results are presented as the mean ± SD and each experiment was performed in triplicate. Statistical differences between compounds and control samples were assessed by one-way or two-way ANOVA followed by Tukey’s post-test performed using GraphPadPrism 6. Statistical differences were considered significant when *P* < 0.05.

## Supplementary Information

Supplementary Information.
