# Supplementary material for: C-Glucosylation as a tool for the prevention of PAINS-induced membrane dipole potential alterations
Source: Sci Rep. 2021 Feb 24;11:4443. doi: 10.1038/s41598-021-83032-3 (PMC7904931; doi:10.1038/s41598-021-83032-3)
Supplement: Supplementary file 1 — Supplementary Information. [file 41598_2021_83032_MOESM1_ESM.pdf]

Supplementary Information

C-Glucosylation as a tool for rescuing PAINS-induced membrane dipole potential alterations

Ana Marta de Matos,[a‡] Maria Teresa Blázquez-Sánchez,[a,b‡] Carla Sousa,[a] Maria Conceição Oliveira,[c] Rodrigo F.M. de Almeida,[a,*] Amélia P. Rauter [a,*]

a. Centro de Química Estrutural, Faculdade de Ciências, Universidade de Lisboa, Ed. C8, Campo Grande, 1749-016 Lisboa, Portugal.

b. Facultad de Ciencias y Artes. Universidad Católica Santa Teresa de Jesús de Ávila (UCAV), 05005 Avila, Spain.

c. Centro de Química Estrutural, Instituto Superior Técnico, Mass Spectrometry Facility, Av. Rovisco Pais, 1049-001 Lisboa, Portugal.

*Corresponding Authors. Contacts: aprauter@fc.ul.pt, rfalmeida@fc.ul.pt;

‡ These authors contributed equally to this work.

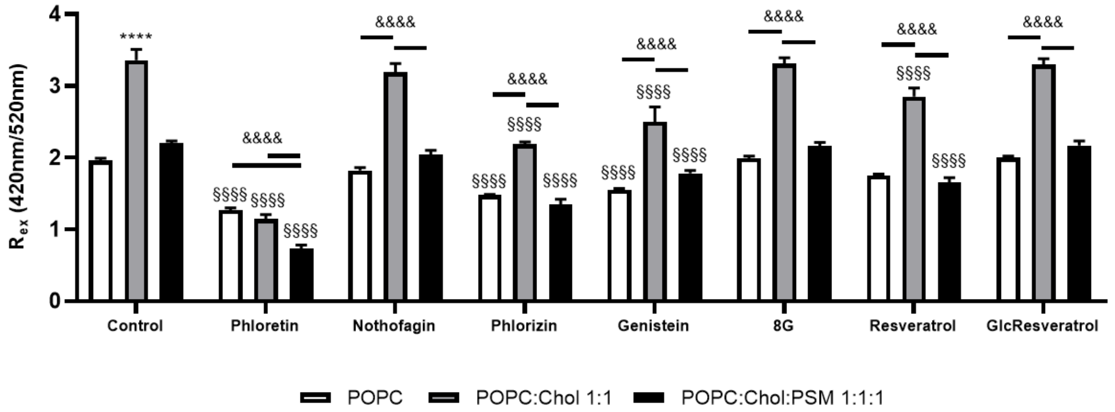

Figure S1. Statistical analysis of membrane dipole potential measurements through the excitation intensity ratio at 420 nm / 520 nm of di-8-ANEPPS for all compounds in all lipid systems**.** Each compound was added to reach a final concentration of 50 µM. Results are presented as the mean ± SD of at least three independent experiments. Statistical differences between compounds and control samples were assessed by two-way ANOVA followed by a Tukey's multicomparison post-test. $^{****}P < 0.0001$ vs. POPC control; $^{\S\S}P < 0.01$, $^{\S\S\S\S}P < 0.0001$ vs. control in the same lipid system; $^{\&}P < 0.05$, $^{\&\&}P < 0.01$, $^{\&\&\&}P < 0.001$, $^{\&\&\&\&}P < 0.0001$ *vs.* the same compound in a different lipid system. 8G – 8-β-D-glucosylgenistein, also abbreviated in the main text as 8-glucosylgenistein; GlcResveratrol – 4-glucosylresveratrol; POPC – 1-palmitoyl-2-oleoyl-*sn*-glycero-3-phosphocholine; Chol – cholesterol; PSM – *N*-palmitoylsphingomyelin.

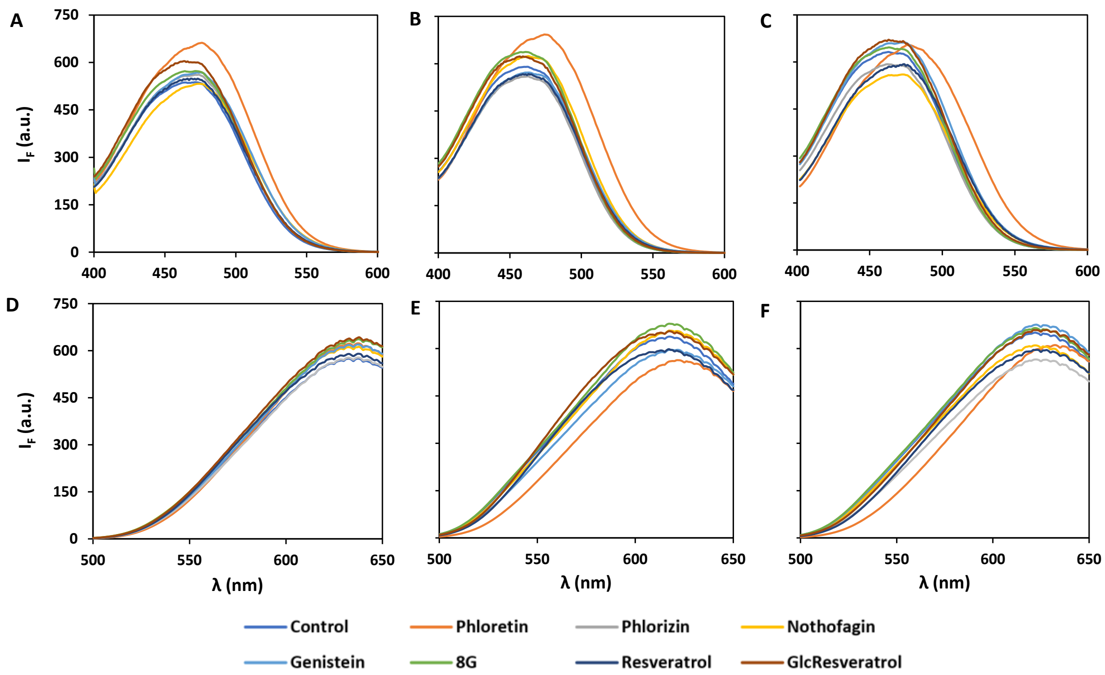

Figure S2. Excitation (A, B, C) and emission (D, E, F) spectra of di-8-ANEPPS in LUV (1 mM total lipid) of POPC (A, D); POPC:Chol (1:1) (B, E) and POPC:Chol:PSM (1:1:1) (C, F) at 23ºC. 1:500 probe: lipid ratio. The spectra were obtained in the absence (control) and presence of different compounds as indicated and are the average of at least three independent replicates. For abbreviations see Figure S1 caption.

The presence of compounds induces small changes in the maximum fluorescence intensity, which are mostly related to the spectral shifts. This is particularly evident for phloretin. For this compound it can be clearly seen that the maximum intensity is usually the highest for the excitation spectrum and the lowest for the emission spectrum. This is due to the spectral shifts occurring in the presence of this compound. Because all the spectra were obtained in the same conditions, the excitation spectrum of phloretin is recorded at an emission wavelength (λ_{em} = 635 nm) that is closer to its maximum and the emission spectrum is recorded at an excitation wavelength (λ_{ex} = 460 nm) that is furthered from its maximum, when compared e.g., to the control. On average, the fluorescence intensity is similar to that of the other samples.

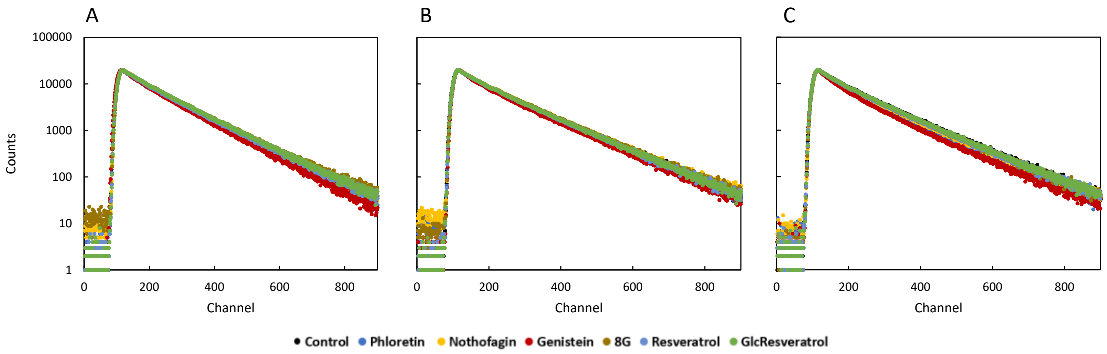

Figure S3. Representative fluorescence intensity decays of DPH-PC obtained by the single-photon timing (SPT) technique in LUV (1 mM total lipid) of POPC (A); POPC:Chol (1:1) (B) and POPC:Chol:PSM (1:1:1) (C) at 23ºC, with a probe: lipid ratio of 1:500, in the absence (control) and presence of different compounds, as indicated. The timescale is 0,055517 ns/channel. In agreement with Fig. 3 in the main text, the fluorescence intensity decay profile for genistein is clearly different (faster decay) from the other samples. For abbreviations see Figure S1 caption.

Table S1. Parameters describing di-8-ANEPPS fluorescence intensity decays in LUV (1 mM total lipid) of POPC; POPC:Chol (1:1) and POPC:Chol:PSM (1:1:1) at 23ºC. 1:500 probe: lipid ratio. All values are the mean ± standard deviation of at least 3 independent measurements. Student's *t*-test significance: $^{*}p < 0.05$, *vs*. control. For abbreviations see Figure S1 caption.

**System**<br>Compound	α_1	τ_1 **(ns)**	α_2	τ_2 **(ns)**	$\bar{\tau}$ **(ns)**	$\langle\tau\rangle$ **(ns)**
**POPC**						
Control	0.21±0.01	1.06±0.13	0.79±0.01	2.10±0.02	1.88±0.01	1.98±0.02
Phloretin	0.41±0.08	1.66±0.11	0.59±0.08	2.61±0.09	2.23±0.13	2.32±0.12
Nothofagin	0.29±0.06	1.32±0.16	0.71±0.06	2.24±0.04	1.99±0.02	2.07±0.02
Phlorizin	0.38±0.02	1.54±0.02	0.62±0.02	2.49±0.02	2.13±0.02	2.23±0.02
Genistein	0.33±0.06	1.38±0.22	0.67±0.06	2.31±0.12	2.01±0.14	2.11±0.12
8G	0.37±0.06	1.32±0.16	0.63±0.06	2.07±0.10	1.80±0.10	1.87±0.09
**POPC:Chol (1:1)**						
Control	0.53±0.12	2.35±0.17	0.47±0.12	3.52±0.16	2.90±0.11	3.01±0.12
Phloretin	0.46±0.06	2.46±0.10	0.54±0.06	3.74±0.09	3.15±0.10	3.28±0.09
Nothofagin	0.53±0.12	2.32±0.20	0.47±0.12	3.56±0.02	2.91±0.04	3.04±0.08
Phlorizin	0.47±0.03	2.36±0.01	0.53±0.03	3.60±0.07	3.02±0.09	3.14±0.09
Genistein	0.52±0.05	2.26±0.12	0.48±0.05	3.52±0.11	2.86±0.07	3.00±0.08
8G	0.53±0.05	2.28±0.07	0.47±0.05	3.39±0.06	2.80±0.01	2.91±0.01
**POPC:Chol:PSM (1:1:1)**						
Control	0.50±0.05	1.86±0.10	0.50±0.05	3.37±0.07	2.61±0.07	2.83±0.06
Phloretin	0.49±0.03	1.67±0.13	0.51±0.03	3.48±0.01	2.59±0.12	2.91±0.06
Nothofagin	0.51±0.03	1.85±0.09	0.49±0.03	3.41±0.08	2.61±0.09	2.85±0.08
Phlorizin	0.49±0.05	1.87±0.14	0.51±0.05	3.47±0.09	2.68±0.09	2.92±0.08
Genistein	0.49±0.04	1.86±0.25	0.51±0.04	3.54±0.05	2.75±0.11	3.00±0.03
8G	0.48±0.02	1.75±0.18	0.52±0.02	3.37±0.05	2.58±0.11	2.84±0.06

Table S2. Parameters describing DPH-PC fluorescence intensity decays in LUV (1 mM total lipid) of POPC; POPC:Chol (1:1) and POPC:Chol:PSM (1:1:1) at 23ºC. 1:500 probe: lipid ratio. All values are the mean ± standard deviation of at least 3 independent measurements. Student's *t*-test significance: *$P < 0.05$, **P < 0.01*vs*. control. For abbreviations see Figure S1 caption.

**System**<br>Compound	α_1	τ_1 **(ns)**	α_2	τ_2 **(ns)**	$\bar{\tau}$ **(ns)**	$<\tau>$ **(ns)**
**POPC**						
Control	0.25±0.05	3.14±0.37	0.75±0.05	7.08±0.05	6.09±0.27	6.57±0.14
Phloretin	0.31±0.06	2.57±0.34	0.69±0.06	6.79±0.09	5.50±0.35	6.19±0.18*
Nothofagin	0.27±0.07	2.73±0.39	0.73±0.07	6.87±0.08	5.73±0.41	6.33±0.24
Genistein	0.36±0.06	2.37±0.35	0.64±0.06	6.40±0.18	4.93±0.44	5.70±0.28*
8G	0.25±0.04	3.07±0.60	0.75±0.04	7.04±0.09	6.04±0.37	6.55±0.15
Resveratrol	0.27±0.05	2.63±0.37	0.73±0.05	6.98±0.06	5.78±0.34	6.44±0.15
GlcResveratrol	0.24±0.05	2.54±0.33	0.76±0.05	6.99±0.06	5.91±0.32	6.53±0.14
**POPC:Chol (1:1)**						
Control	0.25±0.04	2.55±0.20	0.75±0.04	7.34±0.08	6.15±0.25	6.85±0.16
Phloretin	0.30±0.03	2.71±0.38	0.70±0.03	7.20±0.13	5.87±0.20	6.58±0.19
Nothofagin	0.25±0.03	2.59±0.19	0.75±0.03	7.32±0.11	6.13±0.19	6.82±0.15
Genistein	0.35±0.07	2.51±0.09	0.65±0.07	6.97±0.28*	5.43±0.47*	6.25±0.42*
8G	0.25±0.02	2.63±0.21	0.75±0.02	7.35±0.07	6.18±0.14	6.85±0.11
Resveratrol	0.26±0.03	2.54±0.30	0.74±0.03	7.31±0.09	6.07±0.18	6.79±0.13
GlcResveratrol	0.26±0.02	2.51±0.37	0.74±0.02	7.35±0.10	6.10±0.06	6.83±0.13
**POPC:Chol:PSM (1:1:1)**						
Control	0.31±0.03	2.63±0.39	0.69±0.03	7.45±0.15	5.93±0.32	6.78±0.22
Phloretin	0.38±0.04	2.45±0.21	0.62±0.04	7.15±0.15*	5.37±0.31*	6.34±0.27*
Nothofagin	0.33±0.04	2.49±0.28	0.67±0.04	7.34±0.10	5.73±0.19	6.63±0.22
Genistein	0.44±0.06**	2.43±0.18	0.56±0.06	7.16±0.20	5.06±0.33**	6.15±0.34*
8G	0.32±0.05	2.60±0.34	0.68±0.05	7.43±0.18	5.87±0.41	6.74±0.29
Resveratrol	0.32±0.04	2.60±0.33	0.68±0.04	7.42±0.13	5.88±0.33	6.74±0.24
GlcResveratrol	0.31±0.05	2.53±0.35	0.69±0.05	7.43±0.18	5.93±0.41	6.80±0.28

NMR Spectra and bidimensional experiments

Compound 9

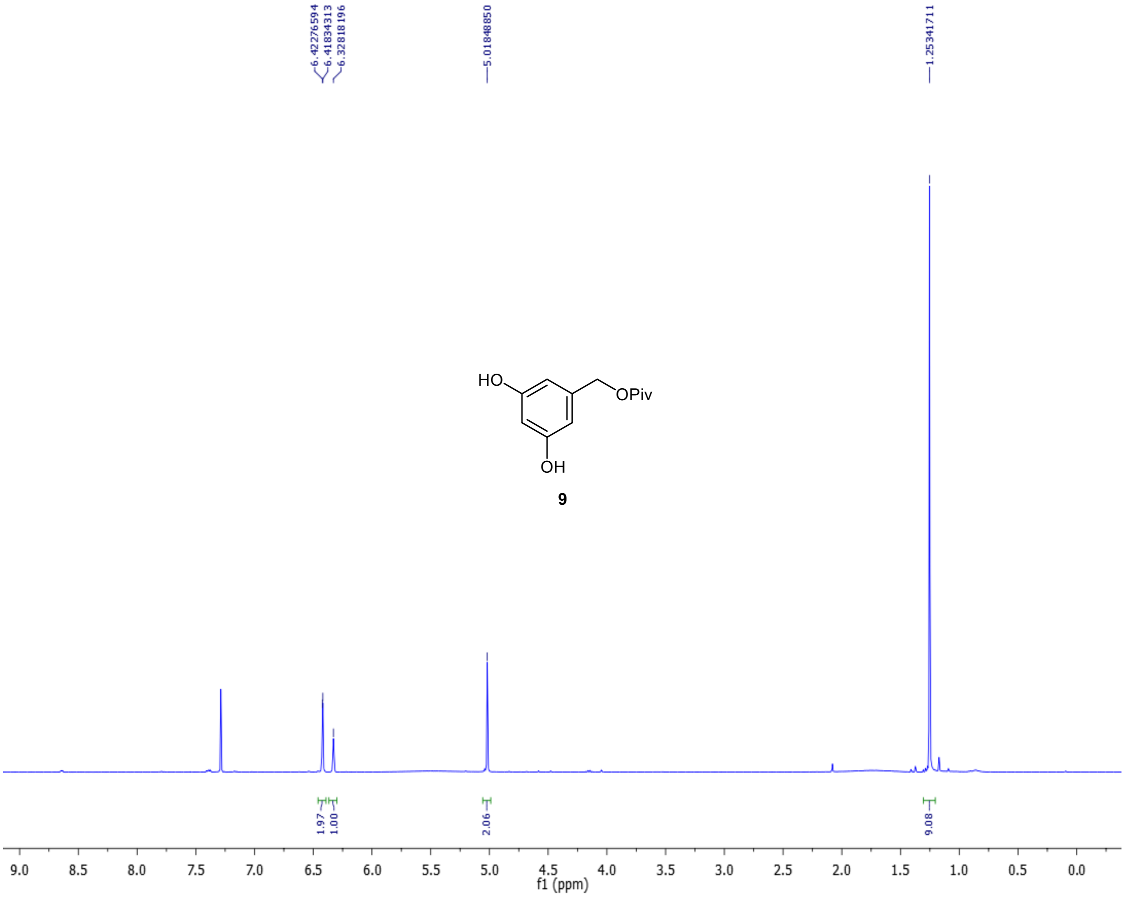

Figure S4. ^{1}H-NMR spectrum of compound **9** in $CDCl_3$.

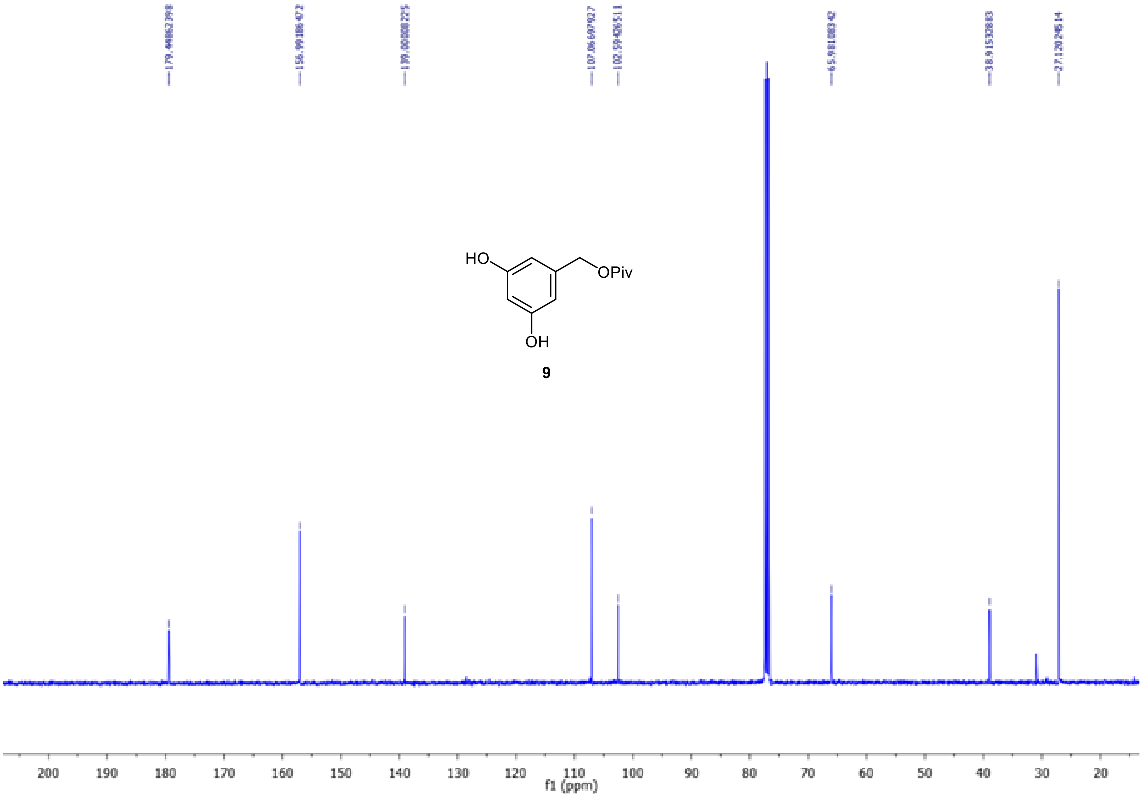

Figure S5. ^{13}C-NMR spectrum of compound **9** in $CDCl_3$.

Compound 10

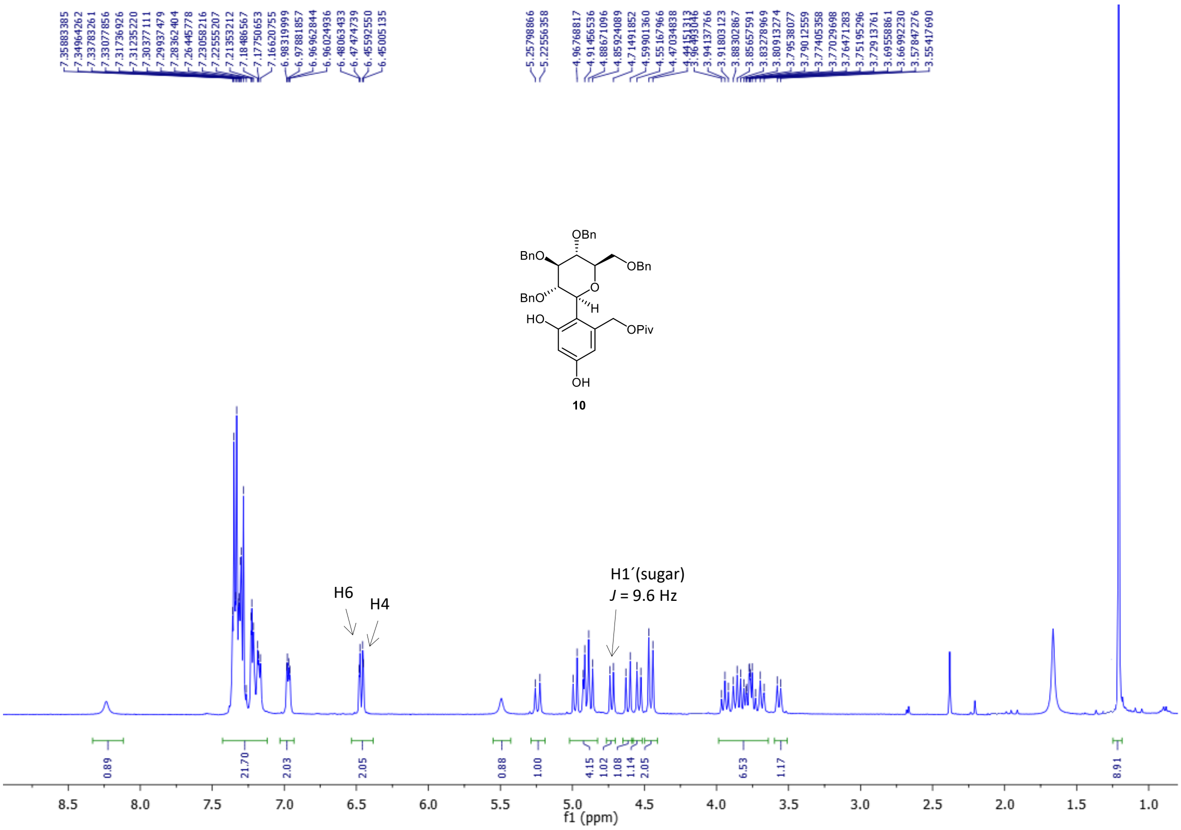

Figure S6. ^{1}H-NMR spectrum of compoun **10** in $CDCl_3$.

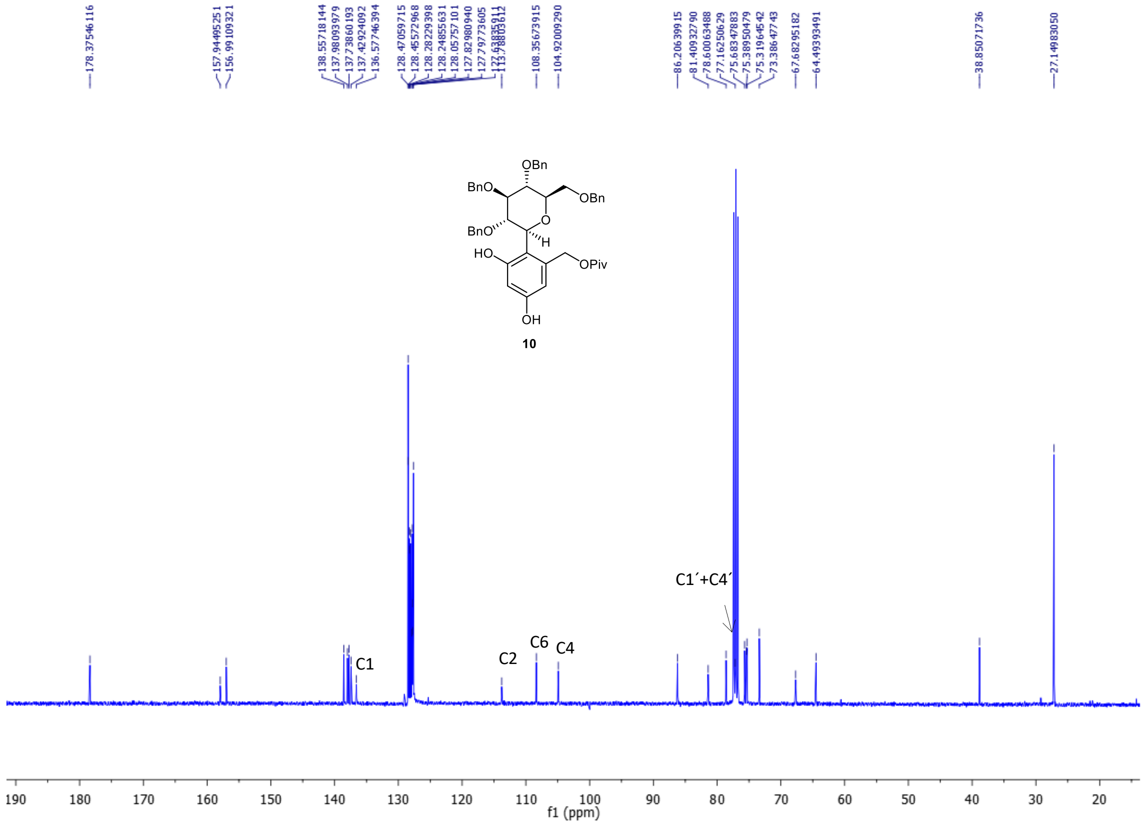

Figure S7. ^{13}C-NMR spectrum of compound **10** in $CDCl_3$.

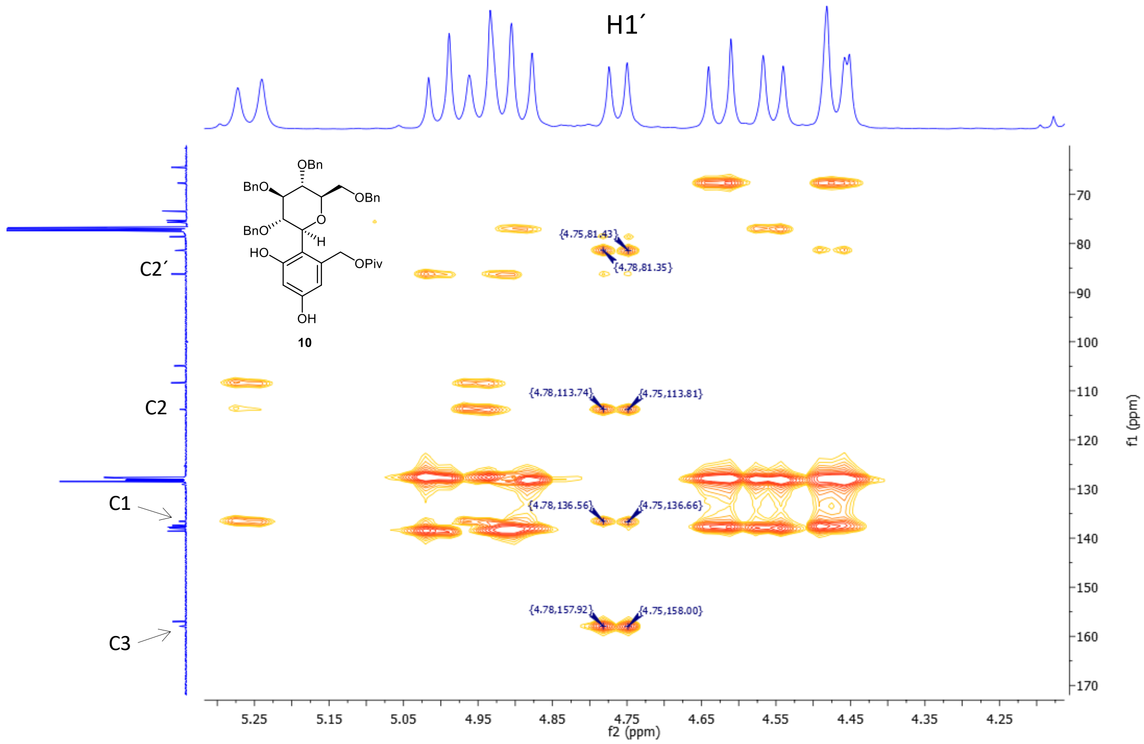

Figure S8. Amplification of the HMBC spectrum of compound **10** in $CDCl_3$. The spectral region shows the correlations of the anomeric proton of the sugar residue H1´ at two and three bond distance with the carbons C1, C2 and C3 of the phenol fragment and C2´ of the sugar unit.

Compound 11

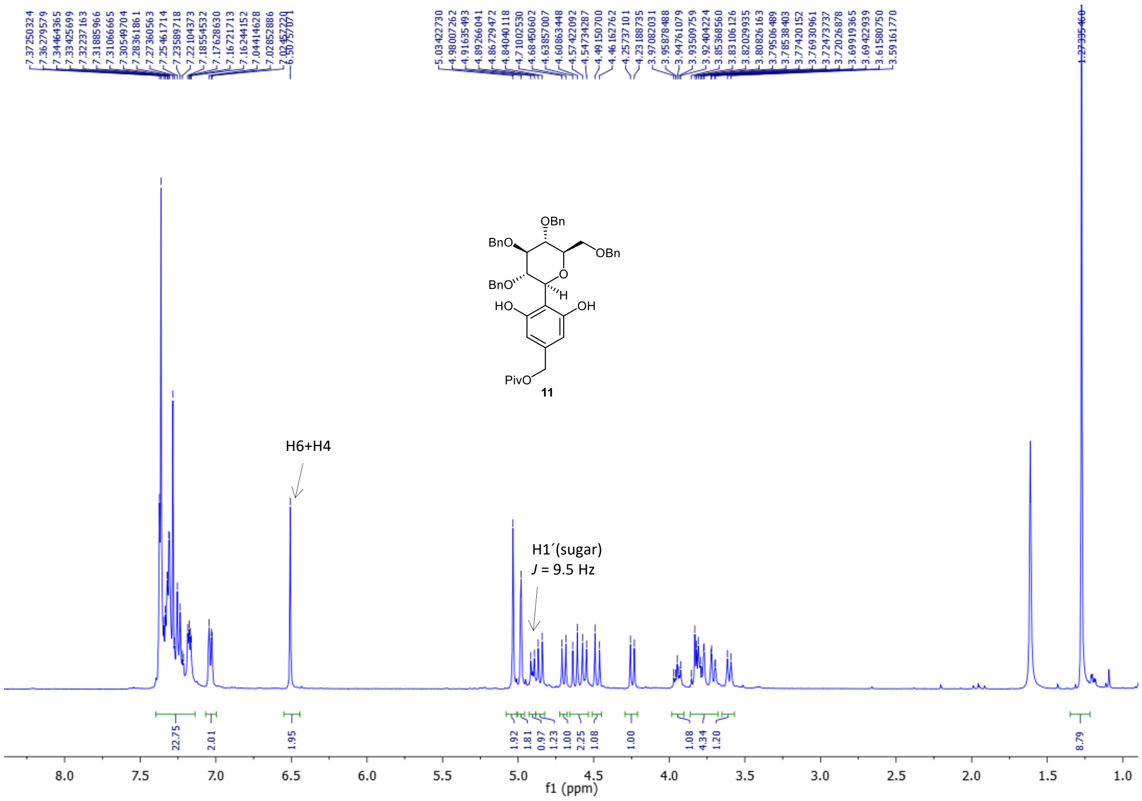

Figure S9. ^{1}H-NMR spectrum of compound **11** in $CDCl_3$.

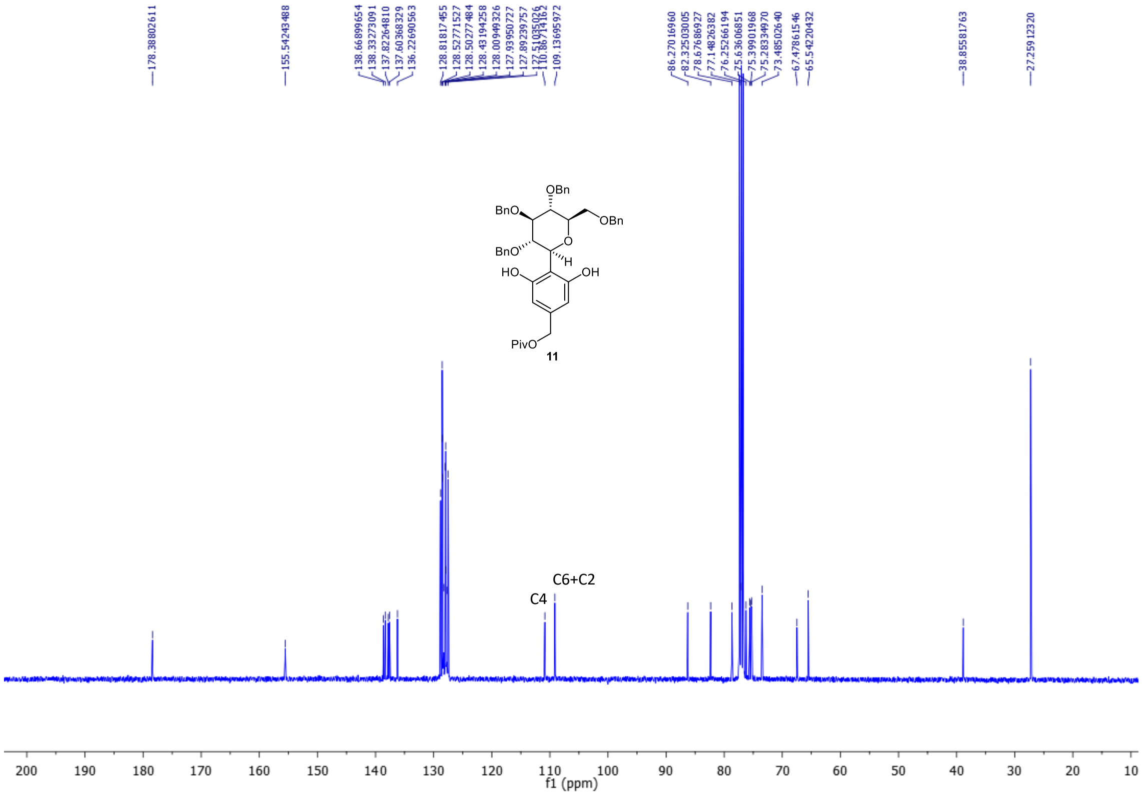

Figure S10. ^{13}C-NMR spectrum of compound **11** in $CDCl_3$

Compound 12

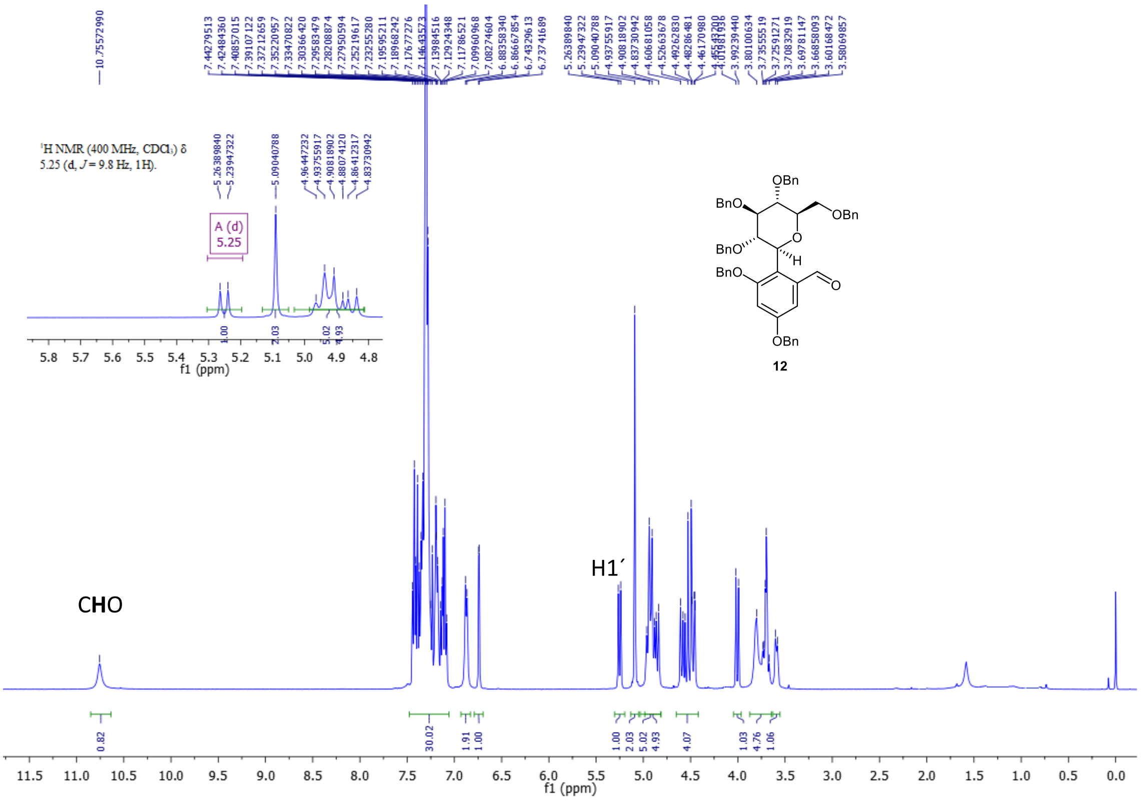

Figure S11. ^{1}H-NMR spectrum of compound **12** in CDCl$_3$.

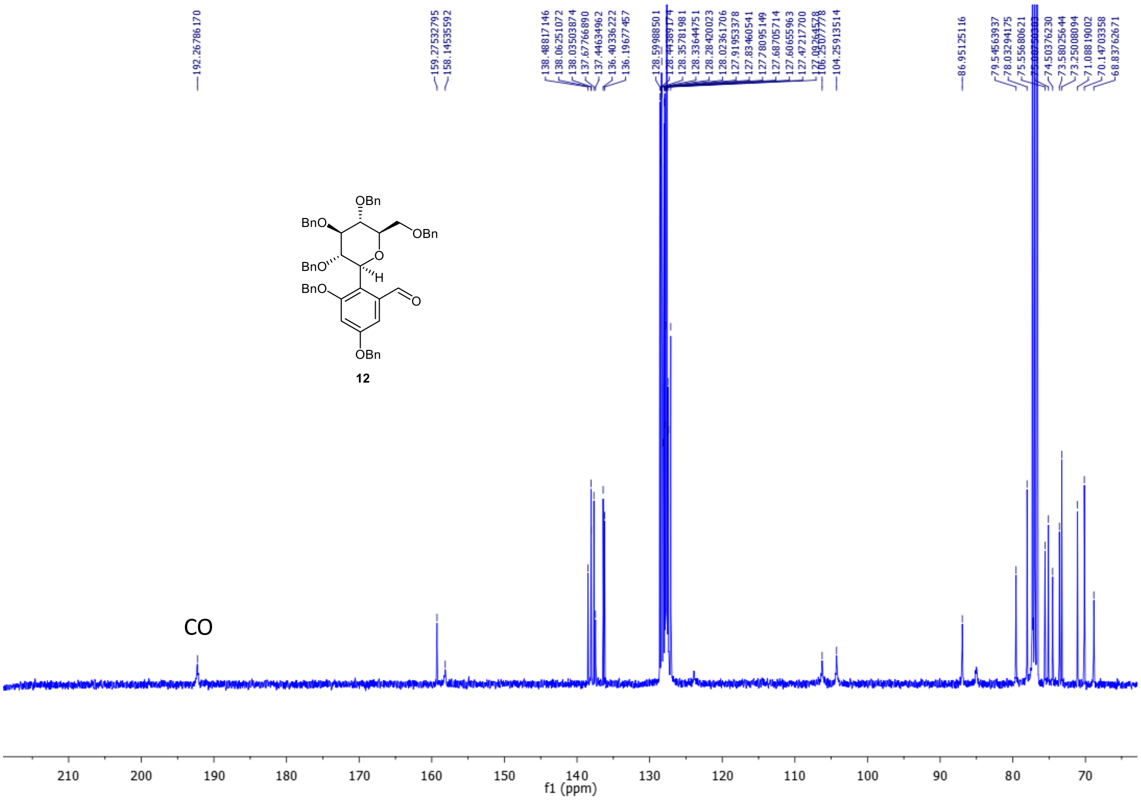

Figure S12. ^{13}C-NMR spectrum of compound **12** in CDCl$_3$.

Compound 14

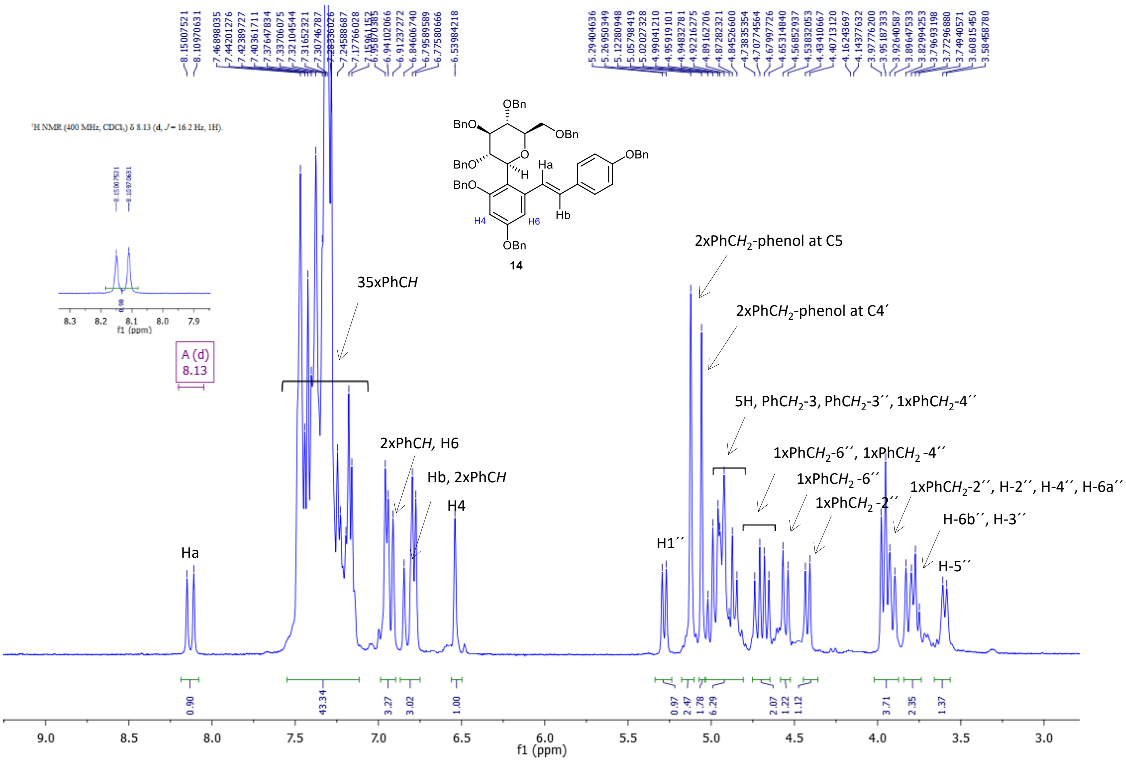

Figure S13. ^{1}H-NMR spectrum of compound **14** in $CDCl_3$.

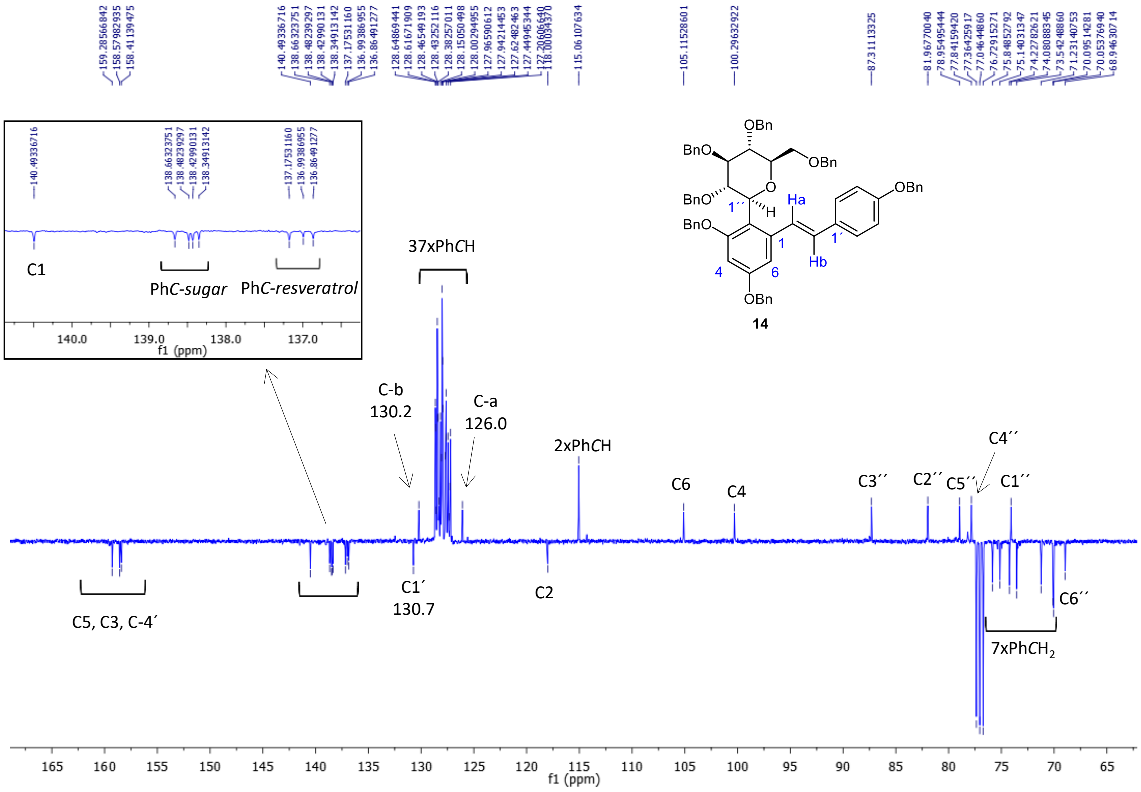

Figure S14. ^{13}C-NMR spectrum of compound **14** in $CDCl_3$.

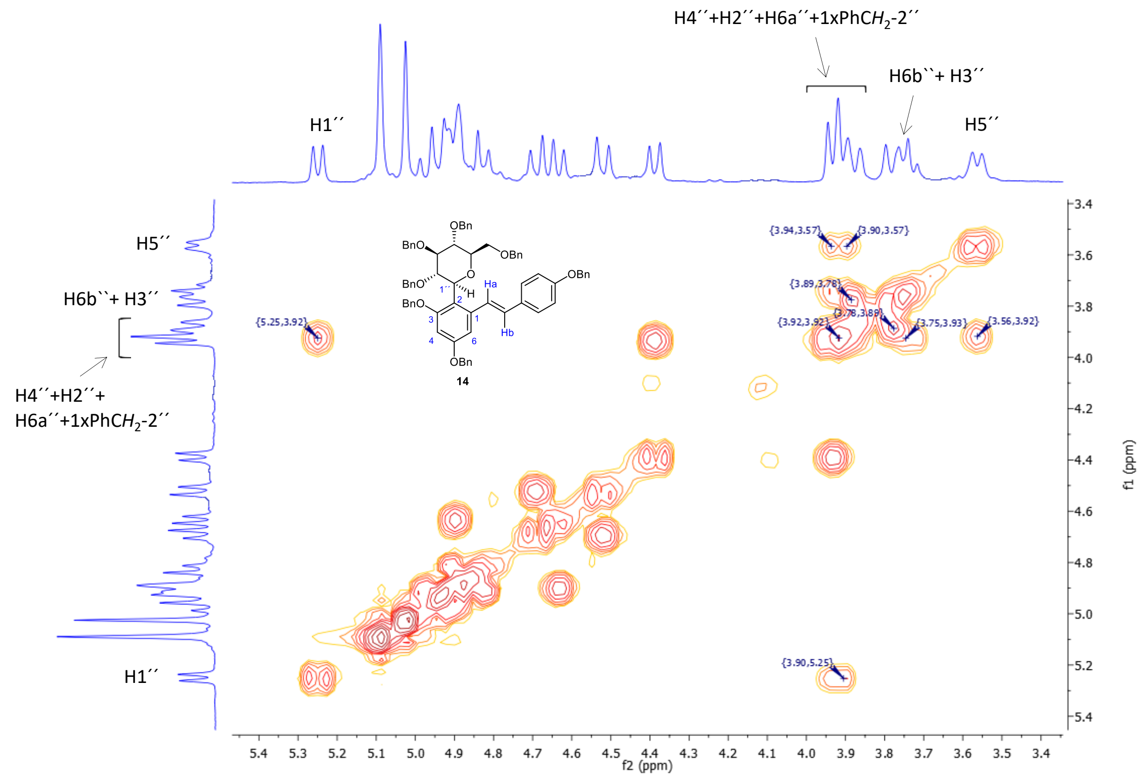

Figure S15. Amplification of the COSY spectrum of compound **14** in $CDCl_3$. The spectral region shows the correlations of the saccharidic protons at three bond distance making possible the assignment of the sugar signals.

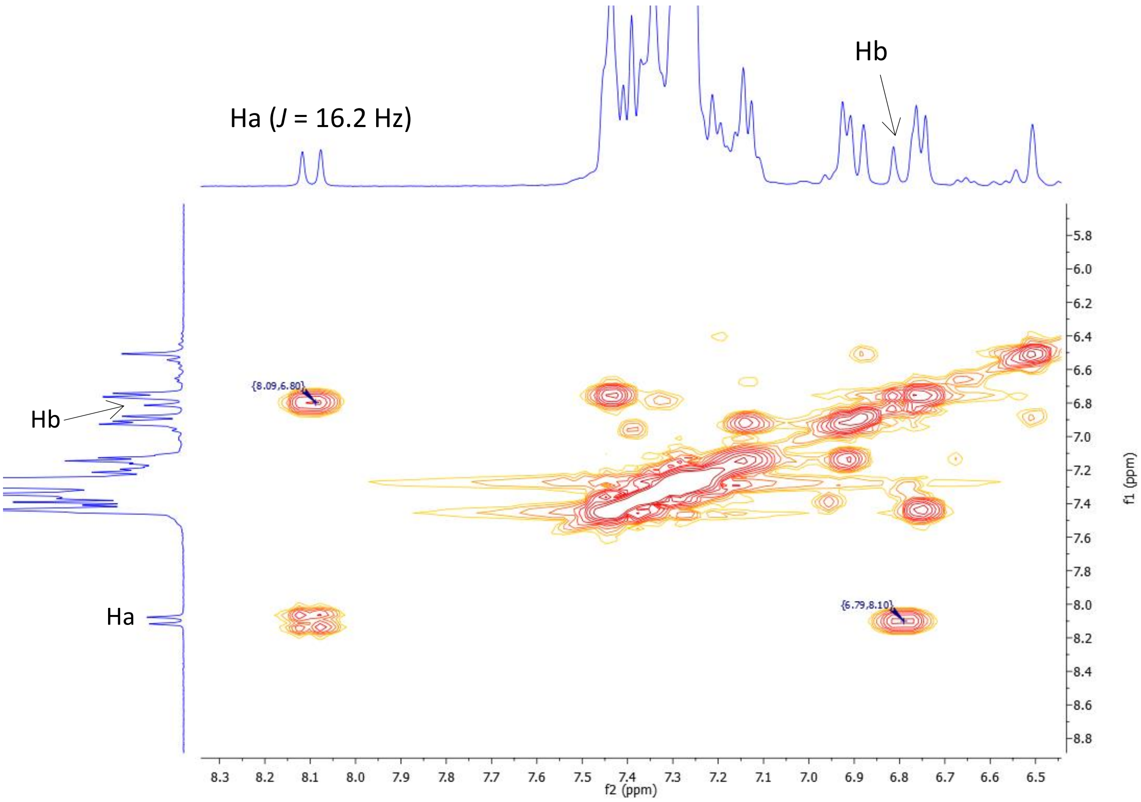

Figure S16. Amplification of the COSY spectrum of compound **14** in $CDCl_3$. The spectral region shows the correlations at three bond distance of the olefinic protons in *trans* configuration (J = 16.2 Hz).

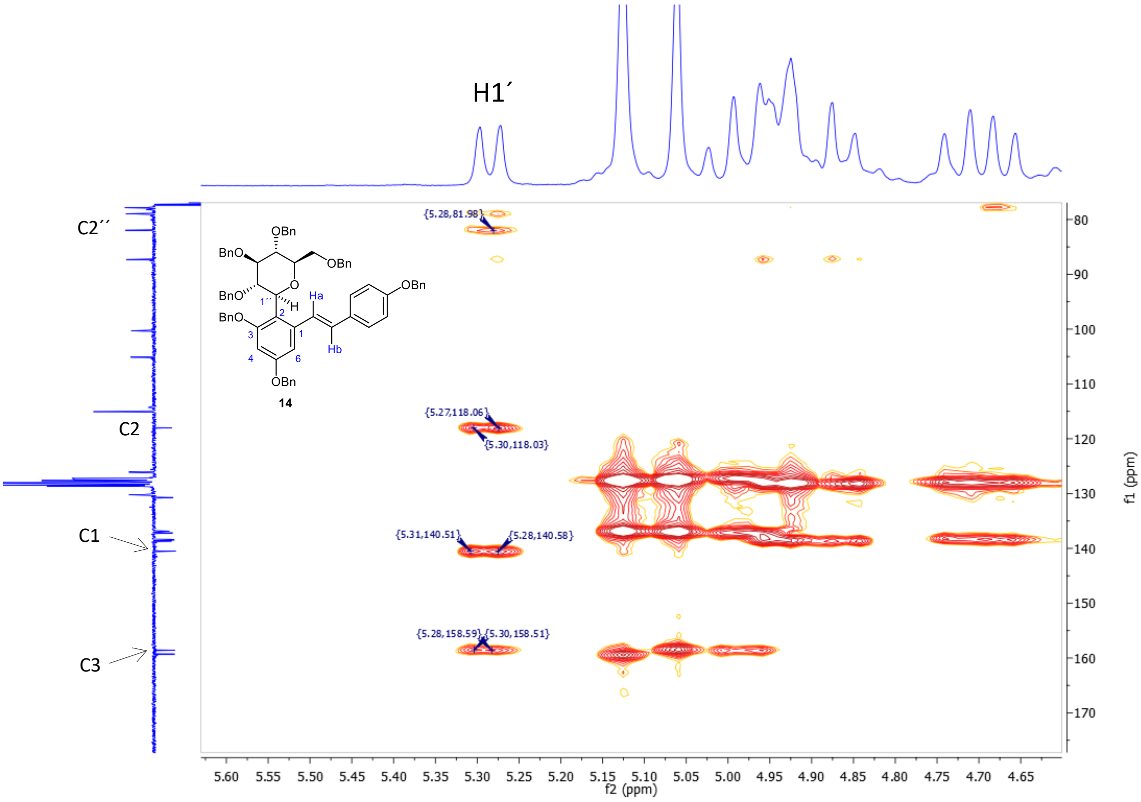

Figure S17. Amplification of the HMBC spectrum of compound **14** in $CDCl_3$. The spectral region shows the correlations of the anomeric proton of the sugar residue H1´´ at two (C1) and three (C2 and C3) bond distance of the phenol fragment and with C2´´ of the sugar unit.

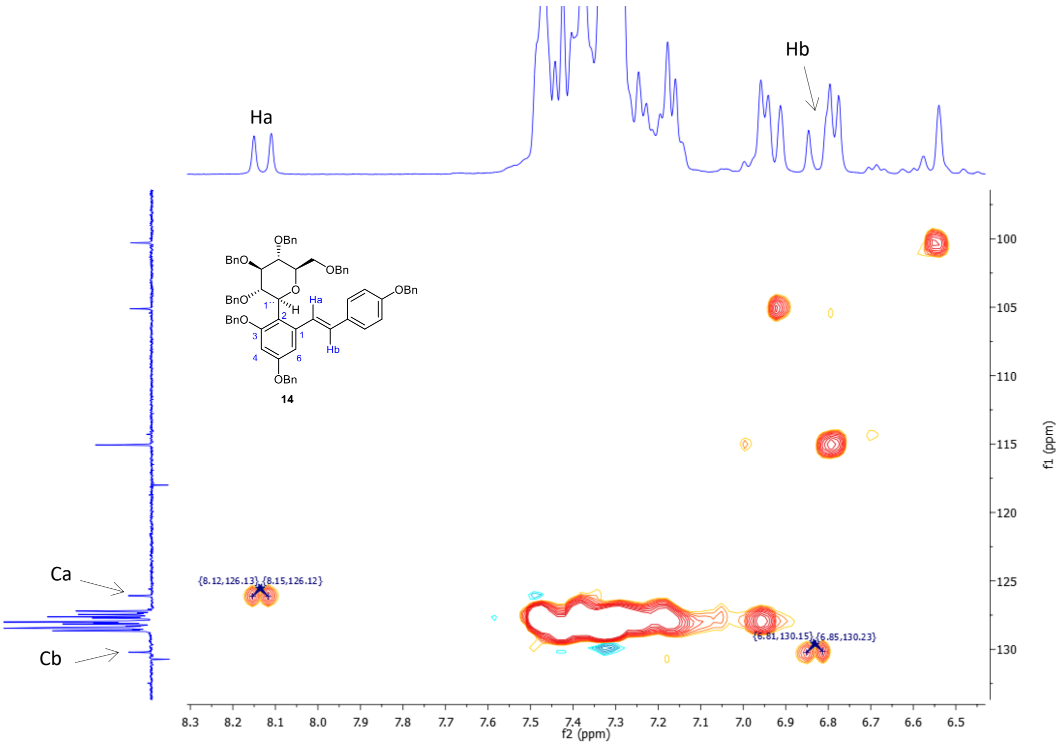

Figure S18. Amplification of the HSQC spectrum of compound **14** in $CDCl_3$. The spectral region shows the correlations of the olefinic protons Ha (8.13 ppm) and Hb (6.82 ppm) with their own carbons Ca and Cb (at 120.0 and 130.2 ppm respectively).

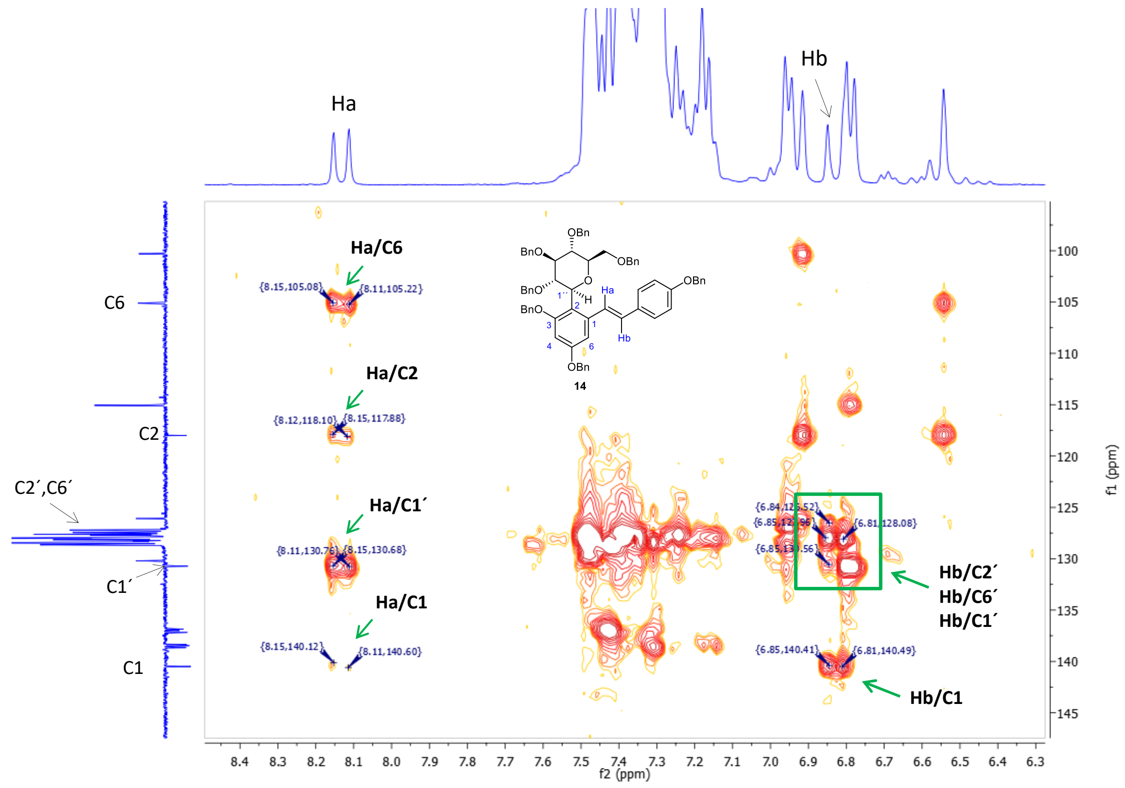

Figure S19. Amplification of the HMBC spectrum of compound **14** in $CDCl_3$. The spectral region shows the correlations of the olefinic protons with carbons C6, C2, C1´, C1 in the case of Ha and C2´,C6´,C1´, C1 with Hb.

Compound 6

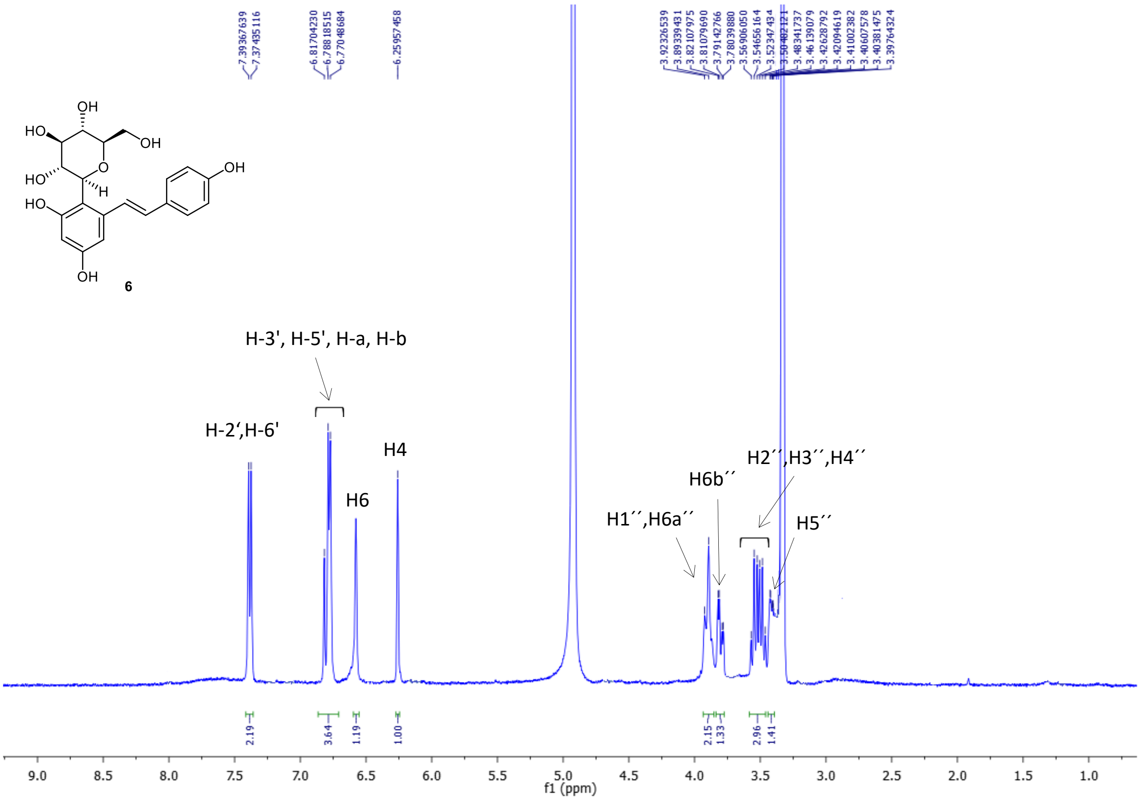

Figure S20. ^{1}H-NMR spectrum of Compound **6** in MeOD

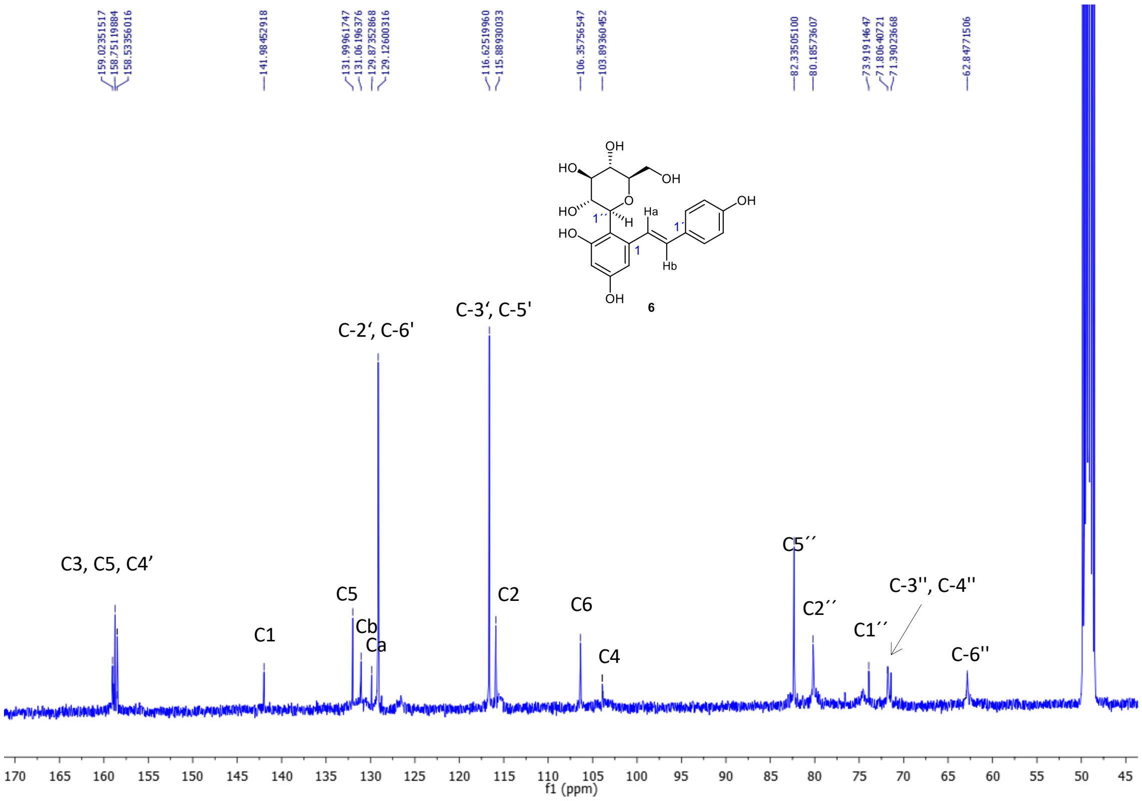

Figure S21.^{13}C-NMR spectrum of Compound **6** in MeOD
